# In a Different Light: Irradiation‐Induced Cuticular Wax Accumulation Fails to Reduce Cuticular Transpiration

**DOI:** 10.1111/pce.15376

**Published:** 2025-01-13

**Authors:** Lena Herzig, Kora Uellendahl, Yaron Malkowsky, Lukas Schreiber, Paul Grünhofer

**Affiliations:** ^1^ Department of Ecophysiology Institute of Cellular and Molecular Botany University of Bonn Bonn Germany; ^2^ Department of Biodiversity of Plants Bonn Institute for Organismic Biology University of Bonn Bonn Germany

**Keywords:** cuticular wax, environmental growth conditions, foliar reflectance, light quantity, plant morphology, residual (cuticular) transpiration

## Abstract

The cuticle, an extracellular hydrophobic layer impregnated with waxy lipids, serves as the primary interface between plant leaves and their environment and is thus subject to external cues. A previous study on poplar leaves revealed that environmental conditions outdoors promoted the deposition of about 10‐fold more cuticular wax compared to the highly artificial climate of a growth chamber. Given that light was the most significant variable distinguishing the two locations, we hypothesized that the quantity of light might serve as a key driver of foliar wax accumulation. Thus, this study aimed to isolate the factor of light quantity (photosynthetic photon flux density [PPFD]) from other environmental stimuli (such as relative humidity and ambient temperature) and explore its impact on cuticular wax deposition and subsequent rates of residual foliar transpiration in different species. Analytical investigations revealed a significant increase in cuticular wax amount with increasing PPFD (between 50 and 1200 µmol m^−2^ s^−1^) in both monocotyledonous (maize and barley) and dicotyledonous (tomato and bean) crop species, without altering the relative lipid composition. Despite the increased wax coverages, rates of foliar water loss did not decrease, further confirming that the residual (cuticular) transpiration is independent of the cuticular wax amount.

## Summary statement

An increase in PPFD (photosynthetic photon flux density) significantly promoted the foliar cuticular wax deposition in four different species, without altering the relative lipid composition. However, increased cuticular wax amounts did not result in lower rates of residual (cuticular) transpiration.

## 1 Introduction

Sunlight is the primary source of energy for plants and thus, ultimately, for all life on earth. Not only light intensity, but also spectral composition, direction and duration influence fundamental plant physiological and morphological processes such as growth, shoot and leaf anatomy, and flowering (Kami et al. 2010; Shafiq et al. 2021). Of the natural spectrum, plants primarily use solar radiation between 400 and 700 nm for photosynthesis which is therefore defined as photosynthetically active radiation (PAR). The quantum of PAR is commonly expressed as photosynthetic photon flux density (PPFD; given in µmol [photons] m^−2^ s^−1^ between 400 and 700 nm). As PAR (light quality) constitutes the main driver of photosynthetic carbon fixation, PPFD (light quantity) critically determines the vegetative growth and yield potential of plants (Corré 1983; Dou and Niu 2020), but their interaction also crucially navigates plant‐internal signalling processes and overall development (Hall and McWatters 2005). In the field, PPFD can vary drastically over the season (summer vs. winter) and even on a daily basis (due to clouding or shading), and is even more dynamic within canopies of individual plants. This poses a considerable challenge for researchers: On a sunny mid‐summer day, maximum PPFD values above 2000 µmol m^−2^ s^−1^ can be reached (Ritchie 2010; Rezai et al. 2018), but only represent a snapshot of the daily light conditions and are hardly representative. Thus, the daily light integral (DLI; PPFD integrated over a day, mol [photons] m ^−2^ d^−1^) is frequently used to describe the summed‐up light intensity experienced by plants (Poorter et al. 2016; Poorter et al. 2019; Grünhofer, Herzig, and Schreiber 2022; Grünhofer, Herzig, Sent, et al. 2022). Numerous studies confirmed that an elevation of PPFD (in a controlled environment) or DLI (in field conditions) increased the accumulation of biomass (Corré 1983; Bruggink 1992; Feng et al. 2019; Ghorbanzadeh et al. 2021; Poorter et al. 2016; Poorter et al. 2019), promoted the deposition of beneficial antioxidants (Dou et al. 2018; Bian, Yang, and Liu 2015), and improved grain yield (Feng et al. 2019; Xie et al. 2019; Dong et al. 2019). However, too much light can create an imbalance between energy supply and consumption, which may result in severe oxidative stress that holds the potential to significantly impair plant productivity and growth (Long, Humphries, and Falkowski 1994). Due to their immobile lifestyle, plants are constantly challenged by an ever‐changing light environment. In response, they developed various anatomical and physiological mechanisms to optimize light capture under low light or shading conditions (Chitwood et al. 2015; Yang et al. 2018) or prevent light stress created by intense irradiation (Shafiq et al. 2021; Sharma et al. 2012).

The first point of contact between sunlight and the plant's surface is the cuticle, a protective layer that covers all primary aerial organs of plants (Kunst and Samuels 2003, 2009). The cuticle emerged together with the invasion of land (Riederer 2006) that exposed formerly aquatic plants to a dehydrating atmosphere, high levels of solar irradiance and harmful ultraviolet (UV) radiation. Besides its delicacy (0.1–10 µm in thickness; Stark and Tian 2006), the cuticular membrane displays a highly efficient barrier against uncontrolled loss of water and dissolved nutrients (Schönherr and Lendzian 1981; Tukey 1966). The hydrophobic nature of the cuticle is established by long‐chain aliphatic lipids known as cuticular waxes that can be (i) embedded into the stability‐providing cutin polymer matrix (intracuticular waxes) and (ii) deposited on its outermost surface as crystalloids or as a smooth film (epicuticular waxes) (Holloway 1994). Besides the restriction of water loss, the cuticle and associated waxes display the first line of defence against environmental threats and fulfil various protective functions, that include (i) water repellence, also referred to as the ‘Lotus effect’, that provides self‐cleaning properties and prevents water retention on the leaf surface (Barthlott and Neinhuis 1997; Koch and Barthlott 2006), (ii) plant–pathogen interactions (Serrano et al. 2014) and (iii) protection against high light, including the reflection of harmful UV radiation (Grant et al. 2003; Grant et al. 1995) and the attenuation of intense PAR levels (Johnson, Richards, and Turner 1983; Holmes and Keiller 2002; Pfündel, Agati, and Zoran 2006). In the face of climate change and the increasingly hostile environment it creates, an understanding of how the cuticle contributes to plant resilience and how cuticle development is influenced by its surroundings becomes increasingly more important.

As the cuticle forms the boundary surface between a plant and its surrounding environment, it is subject to external stimuli (Shepherd and Wynne Griffiths 2006). In a previous study on poplar (*Populus* × *canescens*) leaves, it was observed that ‘harsh’ outdoor conditions (characterized by strong diurnal and seasonal fluctuations of temperature, relative humidity, light quantity and light quality) promoted the deposition of about 10‐fold more wax compared to the highly controlled and artificial environment of a climate chamber while the relative chemical wax composition did not change (Grünhofer, Herzig, Sent, et al. 2022). The published climate recording indicates that during the plant monitoring and harvest period from May to July of 2020, the mean monthly temperature differed by 0.9‐fold, the relative humidity by 1.2‐fold, and the light quantity by 27.3‐fold when comparing outdoor to the climate chamber conditions (Grünhofer, Herzig, Sent, et al. 2022). Thus, the most significant variation between both environments was light intensity, leading to the hypothesis that the quantity of light might be the key driver of cuticular wax accumulation. Indeed, light has been suggested to stimulate the production of wax already half a century ago (Whitecross and Armstrong 1972; Baker 1974; Reed and Tukey 1982), however, the exact nature of this correlation still remains poorly understood. This study aimed to isolate the factor of light quantity as much as possible from the other abiotic variables (e.g., temperature or relative humidity) and investigate how it would influence the cuticular wax amount (and composition) in a number of different crop species (maize, barley, tomato and bean). In addition, we explored whether different light quantities during cuticle development may affect the cuticular transpiration barrier and/or foliar light reflection properties. For this, we cultivated plants under six different levels of PPFD between 50 and 1200 µmol m^−2^ s^−1^, corresponding to a DLI between 3 and 70 mol m^−2^ d^−1^) in an otherwise close‐to‐constant climatic environment. To interpret the results with regard to plant stress level and overall performance under the respective light treatments, the vegetative growth, leaf anatomy and leaf pigmentation were additionally examined.

## 2 Materials and Methods

### 2.1 Plant Material and Cultivation Conditions

**Figure 1:**
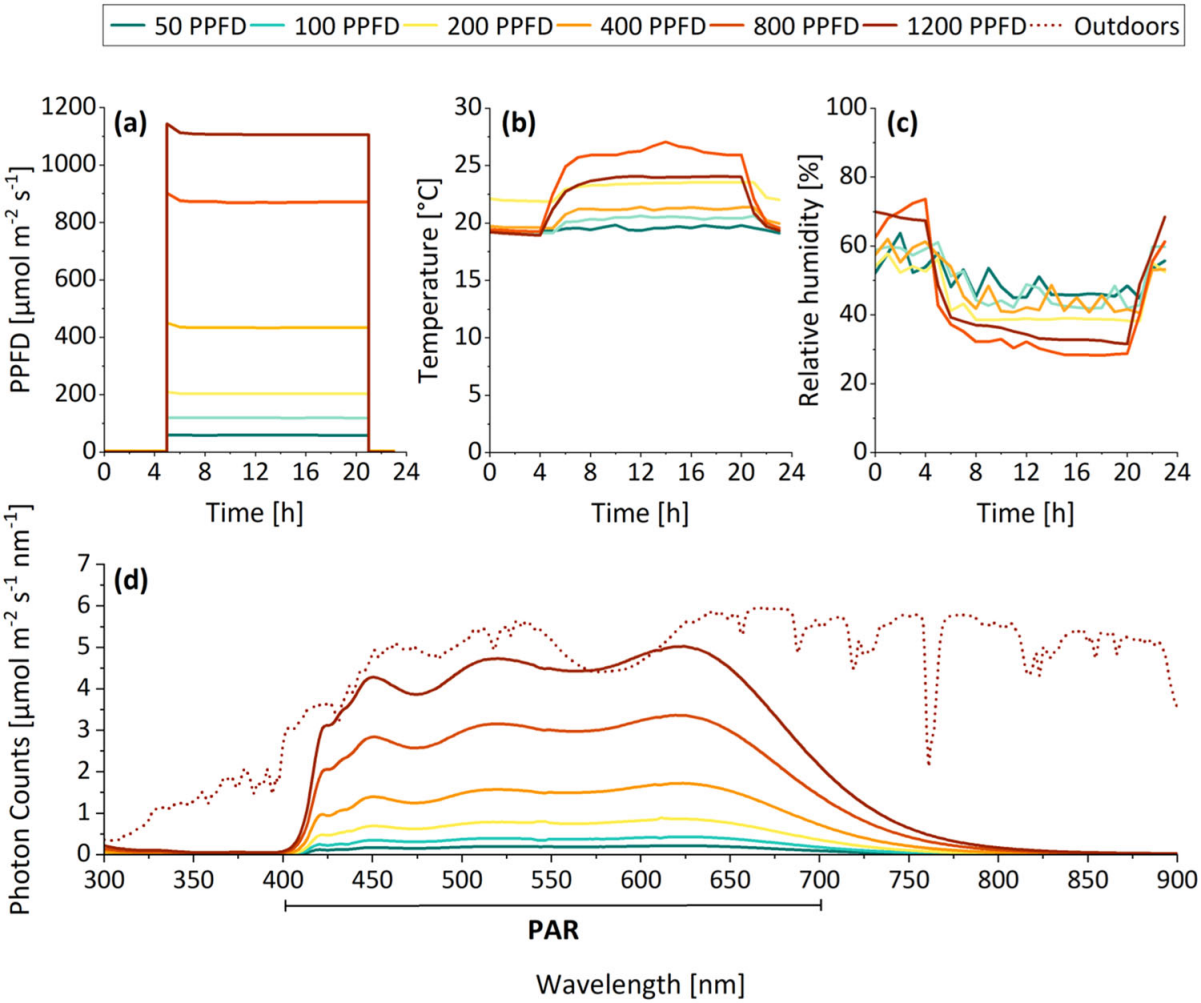
Climatic conditions in the growth chamber shown for one representative day (24 h, illumination/darkness cycle of 16/8 h). (a) The PPFD (light quantity) was set to 50, 100, 200, 400, 800 or 1200 µmol m^−2^ s^−1^. (b) Daily temperature and (c) daily relative humidity were monitored for each light treatment individually. (d) Spectral composition (light quality) of the artificial light sources in the growth chamber (full‐spectrum LEDs, solid lines) in comparison to natural sunlight measured outdoors (dashed line; shows representative data of one sunny afternoon in spring monitored in Bonn, Germany). PAR, photosynthetically active radiation (400–700 nm). PPFD, photosynthetic photon flux density.

Four different crop species of great agricultural and economic value were chosen for this study. Maize (*Zea mays* L.) and barley (*Hordeum vulgare* L., cultivar Bowman) were selected as monocotyledonous plants, whereas tomato (*Solanum lycopersicum* L.) and mung bean (*Vigna radiata* L.) were chosen as dicotyledonous species. The plants were cultivated in a growth chamber with tightly controlled climatic conditions (Figure 1). Six different shelves were used to create six distinct light treatments, each adjusted to a PPFD between 50 and 1200 µmol m^−2^ s^−1^ (see Section 2.2 and Figure 1a). Except for light quantity, the ambient climate was kept as stable as possible. Temperature (Figure 1b) and relative humidity (Figure 1c) were monitored for each light treatment individually using a Minikin QTHi sensor (Environmental Measuring Systems, Czech Republic). During the illumination period (16 h per day), the average temperature varied between a minimum of 19.6°C and a maximum of 25.9°C, while the average relative humidity ranged from 31.4% to 47.3%. During the dark phase (8 h per day), average temperatures were between 19.3°C and 22.0°C, and the average humidity was between 54.3% and 64.7%.

Individuals of all four species were cultivated and harvested (see Section 2.3) in four subsequent cultivation cycles; one for investigations of biomass and leaf morphology (see Section 2.4), one for cuticular wax analysis (see Section 2.5), one for residual transpiration measurements (see Section 2.6), and one for the study of leaf surface properties (see Section 2.7). To ensure consistent cultivation conditions over the entire experimental series, plants were germinated and grown following an identical procedure during each cultivation cycle. A sufficient number of seeds were embedded in paper towels soaked with tap water and incubated in the dark at 25°C for 3 days. The seedlings were carefully placed in pots (1 L volume; four seedlings per pot) filled with standardized soil (Einheitserde Classic Type Topf 1.5, Einheitserde Werksverband e.V., Germany) and placed under the respective light treatment. For each plant species and corresponding light treatment, two pots with eight plants in total were prepared. The pots were watered regularly to avoid dehydration.

### 2.2 Light Treatments

The plants were grown under six different irradiation levels of full‐spectrum light (LED‐KE 400 VSP, DH Licht GmbH, Germany). The PPFD (light quantity) and corresponding spectrum (light quality) were measured using a spectrophotometer (AvaSpec‐ULS2048CLRS‐EVO, Avantes, Netherlands) with an associated light fibre (FC‐UVIR400‐10‐MS‐SMA/FC, Avantes, Netherlands) and the software ‘AvaSoft 8.11’. The PPFD was measured 10 cm above the soil level and set to 50, 100, 200, 400, 800 or 1200 µmol m^−2^ s^−1^ (Figure 1a), which equals a DLI of 3, 6, 12, 23, 46 and 69 mol m^−2^ d^−1^ at a daily illumination period of 16 h, respectively. This enabled a 24‐fold increase in PPFD (50 vs. 1200 µmol m^−2^ s^−1^), while care was taken to maintain an otherwise close‐to‐constant (temperature and relative humidity varied by 1.3‐fold and 1.5‐fold at the most during illumination, respectively) climatic environment (Figure 1b,c). The full‐spectrum LEDs used here simulate the spectral composition of natural sunlight (in the range of PAR, Figure 1d), which improved the comparability of results obtained in situ in the growth chamber to in vivo field conditions. Altogether, this experimental set‐up allowed for the highly focused examination of the impact of light quantity on plant development.

### 2.3 Leaf Sampling

All measurements were conducted after 14 days of growth under the respective light treatment in the case of the monocots (barley and maize) and after 21 days in the case of the dicots (tomato and bean). The first fully developed leaf after the cotyledons was used for analysis whenever possible. If it was too small or already initiated senescence, the second leaf was used instead.

### 2.4 Biomass Accumulation and Leaf Morphology

Selected morphological parameters were examined to monitor shoot and leaf development in response to light quantity. For this, the foliar concentration of chlorophylls, flavonols and anthocyanins was measured using a Force A device (Dualex Scientific, France). Further anatomical leaf characteristics were estimated in the course of residual transpiration measurements (see Section 2.6). To estimate the overall accumulation of above‐ground biomass, the shoots were cut off at the soil surface and the fresh weight (FW) was measured directly using an analytical balance with a resolution of ± 0.01 mg (Sartorius, Germany). Subsequently, the shoots were stored at 60°C for at least 24 h until they were completely dry and reweighed to determine the dry weight (DW). Using the shoot FW and DW, the relative water content (RWC) was calculated following RWC = (FW − DW) FW^−1^.

### 2.5 Cuticular Wax Analysis

The cuticular wax was analyzed using gas chromatography and mass spectrometry. The wax extraction was done following the same protocol for each plant species and light treatment if not stated differently. Broad rim vials (opening 0.3 cm²) were filled with chloroform and 10 µg internal standard (Tetracosane, Fluka, Germany). Leaves were gently placed on the opening, with the desired surface (adaxial or abaxial, both were analyzed separately) facing the vial and were inverted for 10 s. To prevent leaking of the solvent, the leaves were supported with polytetrafluoroethylene plates. Previous extraction kinetics have shown that 10 s is an appropriate time to reliably extract the cuticular wax (intra‐ and epicuticular wax) without damaging the leaf tissue or extracting cell‐internal compounds (Baales, Zeisler‐Diehl, and Schreiber 2021). For the monocots, three spots per leaf were extracted and pooled as one biological replicate. Since it was anticipated based on the literature that tomato (Vogg et al. 2004) and bean (Huang et al. 2020) might have considerably lower foliar wax amounts than maize and barley, the number was increased up to 10 spots per leaf when possible (depending on the leaf surface area) to ensure that the quantity of wax in the sample was sufficient for later detection by gas chromatography. In total, four replicates per species and respective light treatment were harvested. The chloroform was completely evaporated under a gentle stream of nitrogen at 60°C. Samples were resuspended in 250 µL chloroform, spiked with 20 µL BSTFA (*N,O*‐Bis(trimethylsilyl)trifluoroacetamide, Macherey‐Nagel, Germany) and Pyridine (Sigma Aldrich, Germany), and derivatized for 45 min at 70°C. During the process of derivatization polar hydroxyl groups are masked by trimethylsilyl ester and ether formation. Subsequently, 1 µL of the samples was analyzed using a gas chromatograph equipped with on‐column injection following a temperature programme specifically designed for wax samples (Grünhofer, Herzig, Sent, et al. 2022). The quantification was achieved using a gas chromatograph coupled to a flame ionization detector (GC‐FID: 6890 N, Agilent Technologies, USA). Individual peak areas were integrated and referred to the known quantity of the internal standard. For the calculation of wax amount per unit leaf surface area, the amounts estimated with GC‐FID were divided by the extraction area (number of extracted spots per leaf × the vial opening). For the identification of single compounds, a gas chromatograph coupled to a mass spectrometer (GC‐MS: 7890 B, Agilent Technologies, USA) was used. The peaks showing individual fragmentation patterns were assigned to a specific substance using an in‐house database.

### 2.6 Residual (Cuticular) Transpiration

The foliar water loss was monitored gravimetrically following the protocol described in Schuster et al. (2016). Four intact leaves per species and respective light treatment were detached from the plants, the leaves were carefully scanned (CanoScan LiDE 400, Canon, Japan), and the FW was determined. Leaf surface area and FW were later used to calculate the fresh leaf mass per area (LMA). The foliar weight loss was documented at regular intervals (every 10 min within the first 60 min, subsequently every 30 min for up to 5 h). In between measurements, the leaves were stored at 25°C in a sealed plastic box over activated silica gel (2% relative humidity). The permeance *P* (m s^−1^) was calculated following *P* = *F* (*A* × Δ*c*)^−1^, where the flow *F* (g s^−1^) equals the slope between two consecutive weight measurements, *A* (m²) is the area across which transpiration occurred (projected leaf area × 2 to consider the upper and lower leaf surface) and Δ*c* (g m^−3^) is the driving force (concentration gradient of water between the leaf interior and exterior). Since the atmospheric water activity over silica gel is reduced to almost zero, and the water activity within the leaf tissue is assumed unity (Nobel 2009; Schuster et al. 2016), the driving force equals the density of liquid water at 25°C (10^6^ g m^−3^) (Kerstiens 1996; Grünhofer et al. 2023). The incremental liquid‐based permeances calculated from the slope between two consecutive gravimetric measurements were plotted against the corresponding relative water deficit (RWD; incremental weight loss related to the respective leaf FW) to visualize the gradually closing stomata after leaf detachment (as representatively shown in Figure S5). Shortly after leaf detachment (RWD between 0 and 0.1), high incremental permeances indicate high rates of water loss through open stomata. With increasing RWD, the incremental permeances decrease and finally reach a plateau (residual transpiration) that is anticipated to represent the period where stomata are closed to the maximum extent. Foliar residual transpiration during maximum stomatal closure was shown to represent cuticular transpiration in many species (Schuster et al. 2016; Grünhofer, Herzig, Sent, et al. 2022) and thus constitutes an effective method to approximate the cuticular permeance when the isolation of astomatous cuticles is not possible. However, a certain degree of residual water loss through stomata that are not entirely closed cannot be ruled out with certainty in every case, and transpiration measured with intact leaves is thus best referred to as residual (cuticular) transpiration rather than genuine cuticular transpiration (Kerstiens 1996; Burghardt 2003). To characterize the rate of final residual (cuticular) transpiration, all individual incremental permeances lying within the plateauing region of the graph (RWD between 0.05 and 0.5, exact values differed between the individual leaves; see Figure S5 as an example) were pooled.

### 2.7 Leaf Surface Properties

High‐resolution images of leaves developed in selected representative light treatments (100 and 1200 µmol m^−2^ s^−1^) were obtained by scanning electron microscopy (SEM; CLARA, Tescan, Czech Republic) equipped with a secondary electron detector. Segments of 1 cm² were mounted to aluminium sample holders and dried over silica gel for 24 h. The samples were sputter‐coated with 6 nm palladium (Leica EM ACE200, Leica, Germany) and examined at an accelerating voltage of 2 kV.

In addition, the foliar reflectance properties of leaves originating from four selected light treatments (100, 400, 800 and 1200 µmol m^−2^ s^−1^) were investigated using a spectrophotometer (AvaSpec‐ULS2048CLRS‐EVO, Avantes, Netherlands) connected to a combined deuterium and halogen light source (AvaLight‐DH‐S, Avantes, Netherlands) that encompasses the ultraviolet (UV‐B and UV‐A, 280–400 nm) and PAR (400–700 nm) spectrum. First, reference measurements were taken using a 98% (300–700 nm) Spectralon white standard (WS−2, Avantes, Netherlands). All reflection values (white standard and sample surface) were measured at a 45° angle. A minimum of four and a maximum of six leaves were investigated per species and respective light treatment. In the case of barley and maize, reflectance was measured at approximately 50% along the leaf blade. For tomato and bean leaves, reflectance measurements were made at the approximate midpoint of the leaf lamina, avoiding venation as much as possible.

### 2.8 Statistical Analysis

The plot design and the statistical analyses were conducted in OriginPro 21b (OriginLab Corporation, USA). Since the great majority of data sets were normally distributed (Shapiro–Wilk test), one‐way ANOVAs with Fisher's LSD at a significance level of *p* ≤ 0.05 were applied. Significant differences are indicated by differential letters. Lowercase and capital letters were used to facilitate the distinction of two species visualized in the same plot.

## 3 Results

### 3.1 Biomass Accumulation and Leaf Morphology

**Figure 2:**
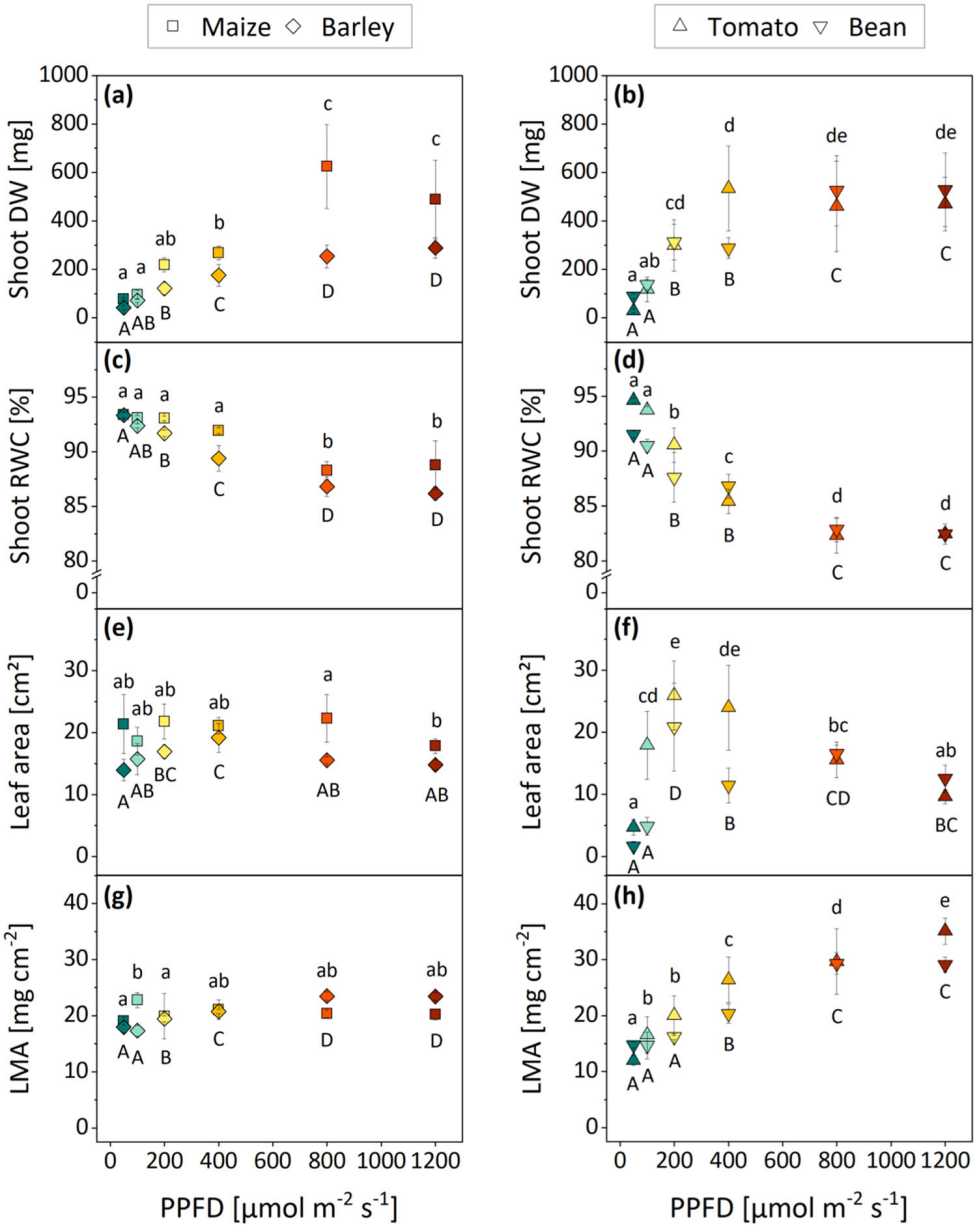
Shoot and leaf morphology of monocots (left) and dicots (right) grown under six different PPFD treatments (colours are based on Figure 1). To assess the overall above‐ground biomass accumulation, shoot dry weight (a, b) and relative water content (c, d) were estimated. In addition, leaf surface area (e, f) and fresh leaf mass per area (g, h) were measured. Means with standard deviations are shown (*n* = 4). Differential letters indicate significant differences (one‐way ANOVA) at *p* ≤ 0.05. Capital and lowercase letters were used to facilitate the distinction between two species in one plot. DW, dry weight; LMA, leaf mass per area; PPFD, photosynthetic photon flux density; RWC, relative water content. [Color figure can be viewed at wileyonlinelibrary.com]

Due to the differences in the rates of height growth and biomass accumulation, the monocots were harvested after 2 weeks of growth, whereas the dicots were analyzed after 3 weeks of growth. A significant increase in shoot DW was observed in all plants as the PPFD was elevated, reaching a maximum under 800 µmol m^−2^ s^−1^ in the case of maize, barley and bean, as well as under 400 µmol m^−2^ s^−1^ in the case of tomato (Figure 2a,b). The increase in shoot DW was accompanied by a corresponding decline in shoot RWC (Figure 2c,d) between −5% (maize and barley) and −10% to −13% (tomato and bean) comparing plants from 50 to 1200 µmol m^−2^ s^−1^. The highest light treatment of 1200 µmol m^−2^ s^−1^ did not result in a further increase in DW (Figure 2a,b) or a further reduction in RWC (Figure 2c,d) compared to 800 µmol m^−2^ s^−1^ in any species.

The leaf morphology of both dicot species responded markedly to PPFD (Figure 2f), while only moderate or even no structural changes were observed in the monocots (Figure 2e). The leaves of tomato and bean remained small when the plants were grown under low PPFD between 50 and 100 µmol m^−2^ s^−1^) but significantly expanded under medium PPFD between 200 and 400 µmol m^−2^ s^−1^). Light quantities exceeding 400 µmol m^−2^ s^−1^ did not result in further increases in leaf size, but rather even led to declines in comparison to the medium PPFD. The LMA continuously increased with PPFD (up to 800 µmol m^−2^ s^−1^ in bean, up to 1200 µmol m^−2^ s^−1^ in tomato) (Figure 2h). In barley, a slight increase in leaf area and LMA was observed comparing the 50–400 µmol m^−2^ s^−1^ treatment, whereas the leaves of maize remained constant in size and relative mass under all light conditions (Figure 2e,g).

**Figure 3:**
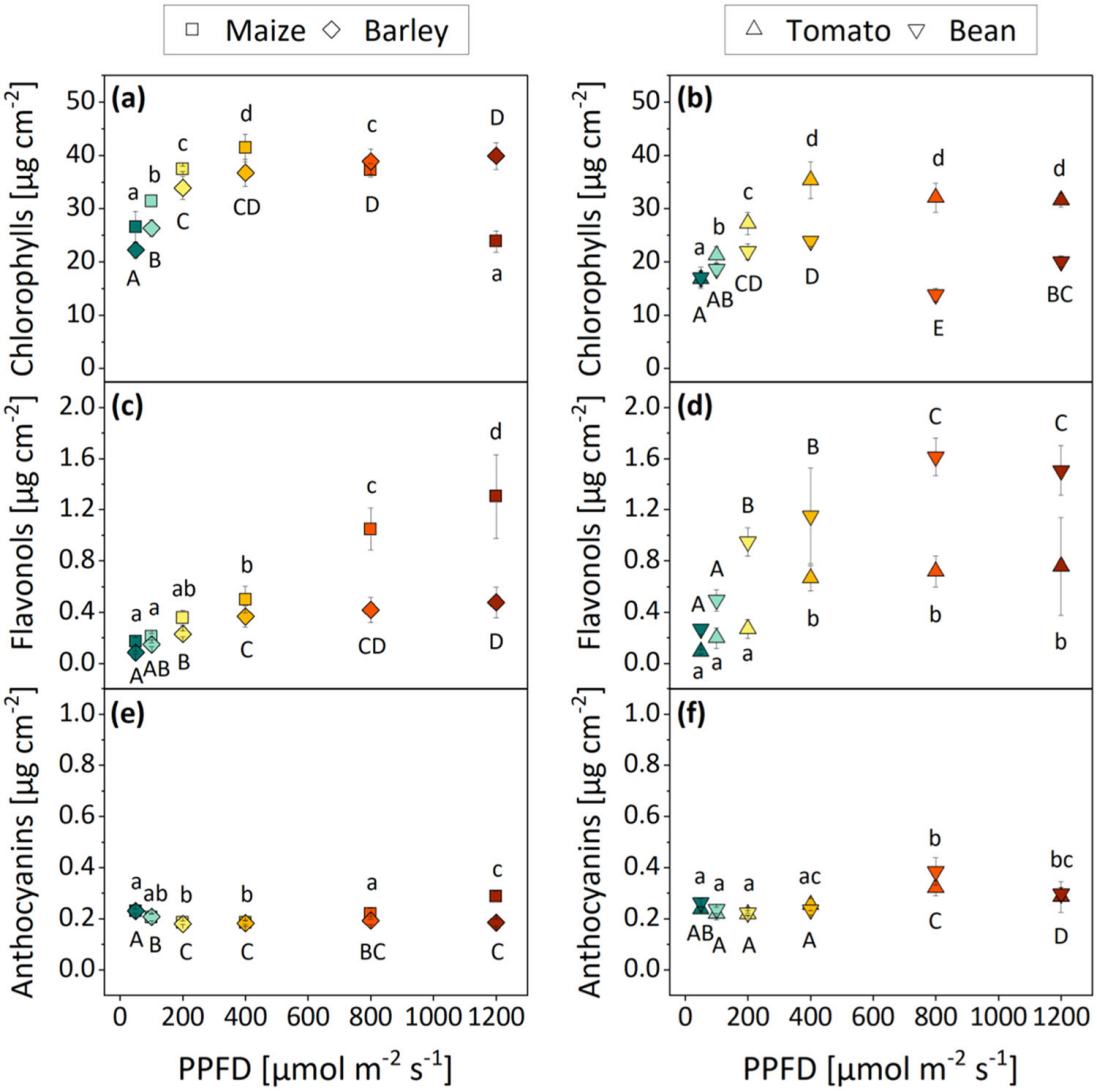
Foliar pigmentation of monocots (left) and dicots (right) grown under six different PPFD treatments (colours are based on Figure 1). The concentration of chlorophylls (a, b), flavonols (c, d) and anthocyanins (e, f) was measured. Means with standard deviations are shown (*n* = 4). Differential letters indicate significant differences (one‐way ANOVA) at *p* ≤ 0.05. Capital and lowercase letters were used to facilitate the distinction between two species in one plot. PPFD, photosynthetic photon flux density. [Color figure can be viewed at wileyonlinelibrary.com]

In addition to the structural characteristics of leaves developed under different PPFD treatments, the influence of light quantity on pigmentation was investigated. Chlorophyll contents increased in all species comparing plants from 50 to 400 µmol m^−2^ s^−1^, and the amount of chlorophyll either stagnated (barley and tomato) or decreased (maize and bean) under high PPFD between 800 and 1200 µmol m^−2^ s^−1^) compared to 400 µmol m^−2^ s^−1^ (Figure 3a,b). Similarly, flavonol contents in barley and tomato moderately increased comparing plants from 50 to 400 µmol m^−2^ s^−1^, while no further accumulation was observed under PPFD > 400 µmol m^−2^ s^−1^ (Figure 3c,d). Maize and bean displayed a substantially higher enrichment in flavonols (approx. threefold more than in barley and tomato) under high PPFD (Figure 3c,d). Maize was the only species where the acceleration of flavonols followed a linear trend with increasing PPFD up to 1200 µmol m^−2^ s^−1^ (Figure 3c). The anthocyanin content varied comparatively little in all four species under the different light conditions (Figure 3e,f).

### 3.2 Cuticular Wax Amount and Composition

**Figure 4:**
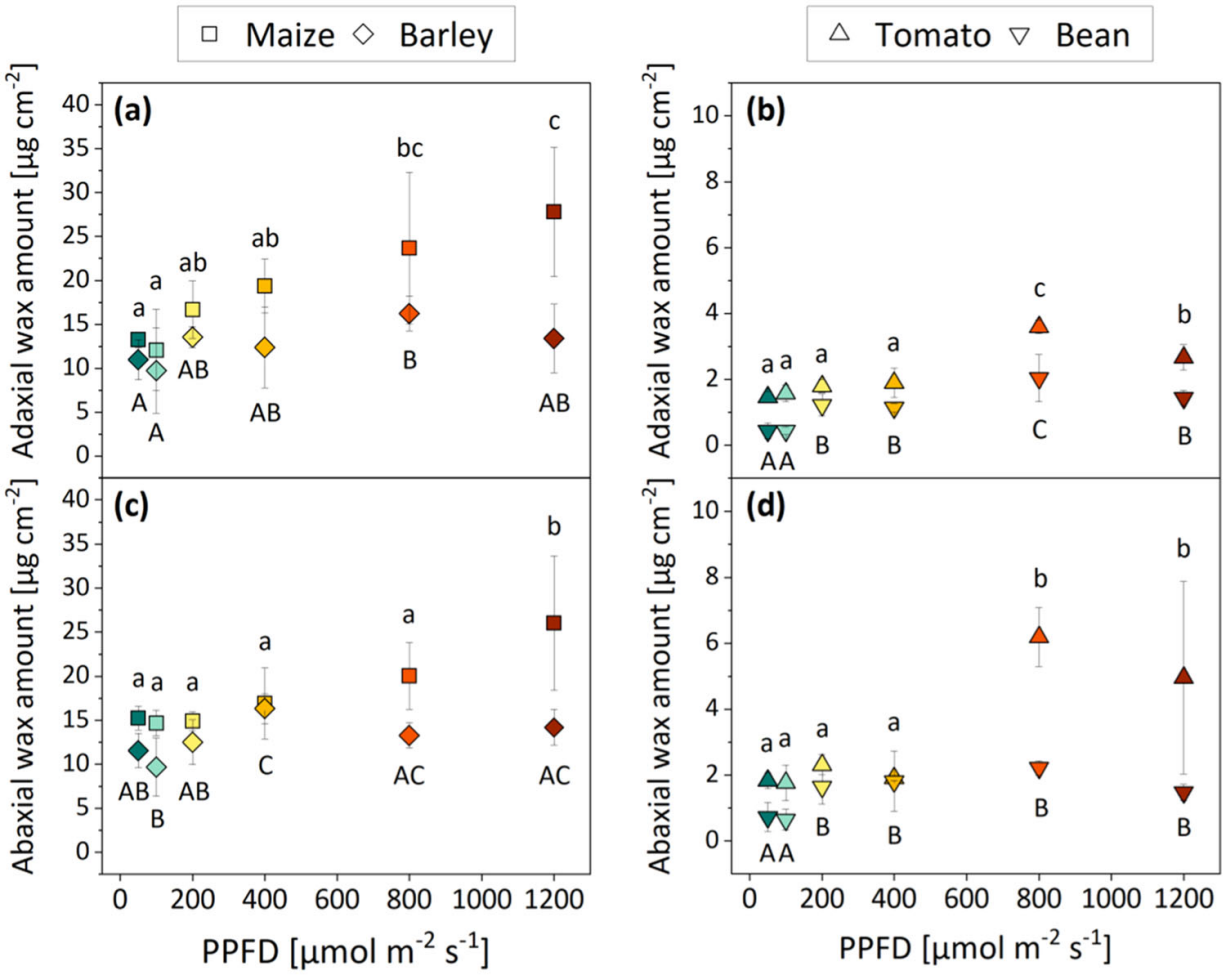
Absolute cuticular wax amount of monocots (left) and dicots (right) grown under six different PPFD treatments (colours are based on Figure 1). The adaxial (a, b) and abaxial (c, d) wax amounts were investigated separately. Means with standard deviations are shown (*n* = 3–4). Differential letters indicate significant differences (one‐way ANOVA) at *p* ≤ 0.05. Capital and lowercase letters were used to facilitate the distinction between two species in one plot. PPFD, photosynthetic photon flux density. [Color figure can be viewed at wileyonlinelibrary.com]

A positive correlation between light quantity and the aliphatic wax amount per unit area was observed in all four species (Figure 4). To assess the impact of PPFD on the adaxial and abaxial leaf surfaces individually, the cuticular wax from both surfaces was extracted and analyzed separately. The reported wax amounts on the adaxial and abaxial surfaces of a selected species were found to be highly similar in most cases (compare Figure 4a with Figure 4c and Figure 4b with Figure 4d). Overall, the monocots had notably more wax (10–30 µg cm^−2^) than the dicots (mostly < 5 µg cm^−2^) (compare Figure 4a with Figure 4b and Figure 4c with Figure 4d). All species exhibited the lowest wax amounts under the lowest PPFD that was tested (50 µmol m^−2^ s^−1^).

The highest wax quantities were found in maize, which simultaneously was the only species that exhibited a linear increase in wax coverage with PPFD even up to the highest treatment of 1200 µmol m^−2^ s^−1^. The wax amount on maize leaves grown under 1200 µmol m^−2^ s^−1^ was twofold higher compared to those from the 50 µmol m^−2^ s^−1^ treatment (approx. 15 vs. 30 µg cm^−2^, Figure 4a,c). In barley, the correlation between wax deposition and light quantity could also be observed, however, not nearly as pronounced as in maize. The cuticular wax amount was 1.4‐fold higher on leaves exposed to PPFD between 400 and 800 µmol m^−2^ s^−1^ compared to 50 µmol m^−2^ s^−1^ (approx. 11 vs. 15 µg cm^−2^, Figure 4a,c). Here, light conditions > 800 µmol m^−2^ s^−1^ did not further increase the accumulation of waxy lipids but resulted in quantities similar to 400–800 µmol m^−2^ s^−1^.

Both dicot species deposited the highest wax amount under 800 µmol m^−2^ s^−1^, while 1200 µmol m^−2^ s^−1^ even resulted in decreased wax coverages compared to leaves obtained from the 800 µmol m^−2^ s^−1^ treatment (Figure 4b,d). In tomato, a PPFD of 800 µmol m^−2^ s^−1^ resulted in 2.3‐fold higher wax amounts compared to 50 µmol m^−2^ s^−1^ (approx. 1.5 vs. 3.5 µg cm^−2^). Bean plants grown under 800 µmol m^−2^ s^−1^ deposited more than fourfold higher wax amounts than those from 50 µmol m^−2^ s^−1^ (approx. 0.5 vs. 2.1 µg cm^−2^).

While there was clear evidence for a correlation between light quantity and the amount of wax that is deposited, the wax composition did not change as considerably (Figures S1 and S2; for specific monomer patterns, see Figures S3 and S4). Only a few significant trends indicating a light‐related relative shift of some functional groups were observed on the abaxial leaf surface of maize and bean but not barley or tomato (Tables S1 and S2), while the predominantly light‐exposed adaxial side of all species lacked any obvious qualitative changes. Thus, the explicit focus was laid on the striking light‐induced increases in wax amount.

### 3.3 Residual (Cuticular) Transpiration

**Figure 5:**
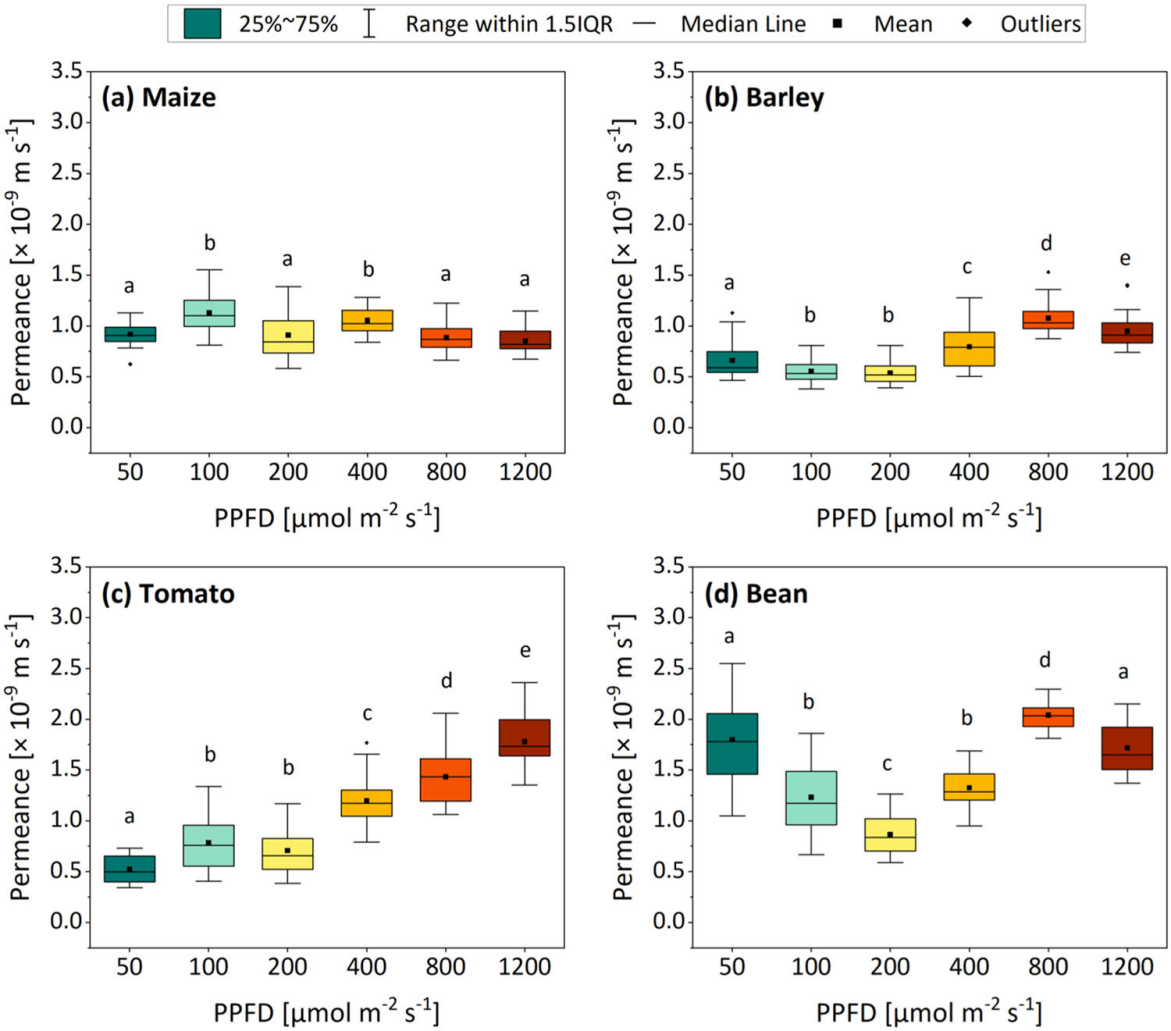
Residual permeances of maize (a), barley (b), tomato (c) and bean (d) plants grown under six different PPFD treatments (colours are based on Figure 1). Boxplots are based on incremental plateauing permeances measured at a relative water deficit between 0.05 and 0.5, which was determined for each measured leaf individually (*n* = 24–40) (an example is visualized in Figure S5). Differential letters indicate significant differences (one‐way ANOVA) at *p* ≤ 0.05. PPFD, photosynthetic photon flux density. [Color figure can be viewed at wileyonlinelibrary.com]

Boxplots shown in Figure 5 were obtained from incrementally calculated permeances that were found to plateau during the period of maximum stomatal closure (as described in Section 2.6; representative plots obtained from the 1200 µmol m^−2^ s^−1^ treatment are shown in Figure S5). No clear correlation between the residual (cuticular) transpiration and the ambient light intensity during plant growth was observed in maize (Figure 5a), barley (Figure 5b) or bean (Figure 5d). In tomato (Figure 5c), the residual permeance increased significantly with increasing PPFD. All mean residual permeances (four species grown under six different light intensities) were found to lie within 0.5–2 × 10^−9^ m s^−1^.

### 3.4 Leaf Surface Properties

**Figure 6:**
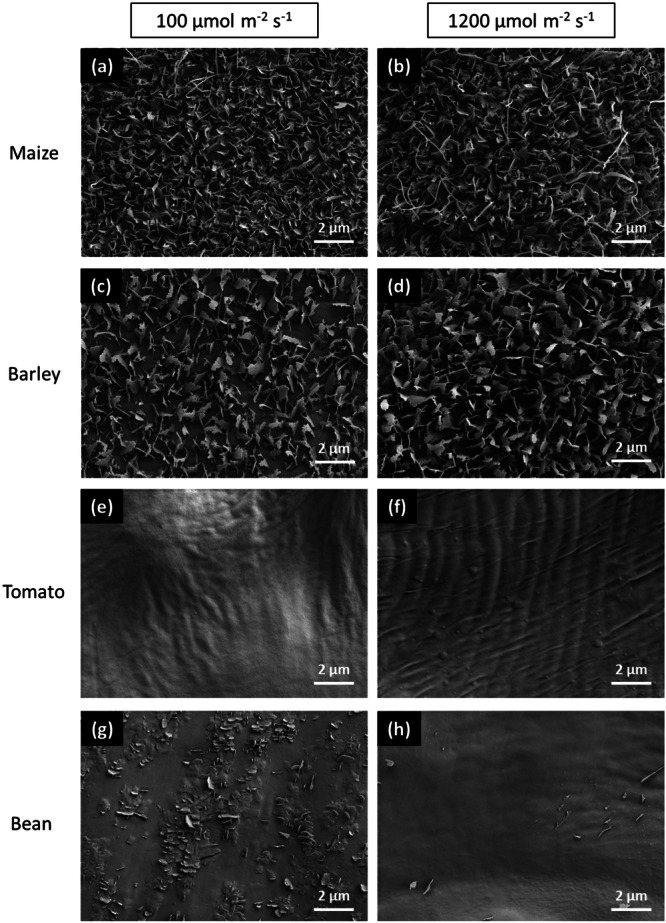
Scanning electron microscopy images of the adaxial leaf surface of maize (a, b), barley (c, d), tomato (e, f) and bean (g, h) plants grown under light quantities of 100 µmol m^−2^ s^−1^ (left) or 1200 µmol m^−2^ s^−1^ (right).

In addition to the cuticular wax analysis and transpiration measurement, the adaxial surface topography (two selected treatments: 100 and 1200 µmol m^−2^ s^−1^) and reflectance properties (four selected treatments: 100, 400, 800 and 1200 µmol m^−2^ s^−1^) of leaves from the four species were studied. High‐resolution images obtained by SEM revealed that the adaxial surface of the monocotyledonous maize and barley leaves was covered by platelet‐shaped epicuticular wax crystals that remarkably increased in density on leaves grown at 1200 µmol m^−2^ s^−1^ compared to 100 µmol m^−2^ s^−1^ (Figure 6a–d). Tomato leaves from both light conditions were entirely void of visible wax crystals (Figure 6e,f). In bean, some crystalloid formations were found in low abundance on the leaves grown under 100 µmol m^−2^ s^−1^ (Figure 6g), while the leaves obtained from the 1200 µmol m^−2^ s^−1^ treatment were almost completely void of wax crystals (Figure 6h).

**Figure 7:**
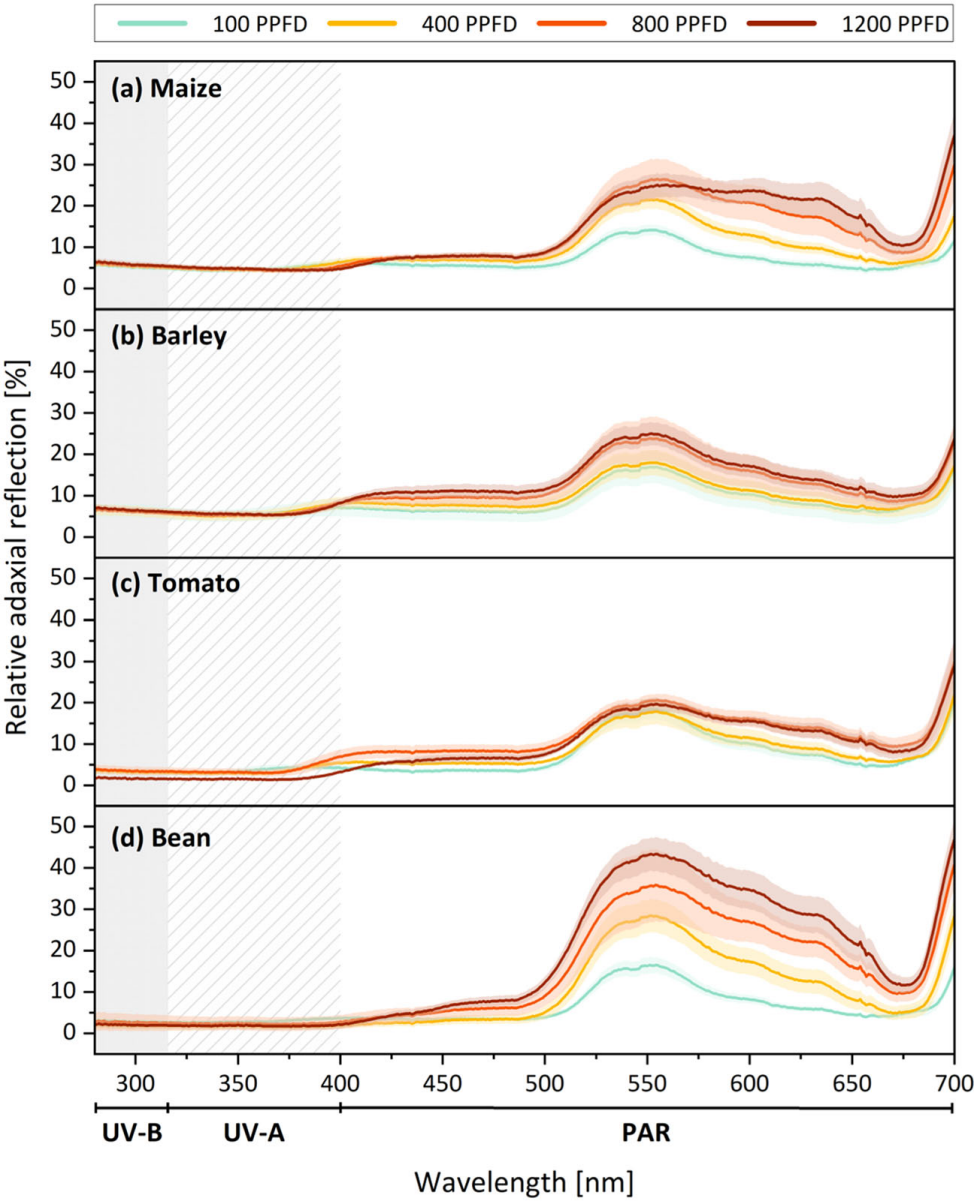
Relative reflection of the adaxial leaf surface of maize (a), barley (b), tomato (c) and bean (d) plants grown under four different PPFD treatments (colours are based on Figure 1). The foliar reflection was investigated in the spectral range between 280 and 700 nm, which comprises reflection in the UV‐range (UV‐B 280–320 nm, UV‐A 320–400 nm) as well as in the PAR range (400–700 nm). Standard deviations around the means are shown as shaded (*n* = 4–6). PAR, photosynthetically active radiation; PPFD, photosynthetic photon flux density; UV, ultraviolet. [Color figure can be viewed at wileyonlinelibrary.com]

The adaxial foliar reflection overall increased in the PAR range when the plants were cultivated under high‐light conditions (Figure 7). This effect was particularly pronounced in bean (Figure 7d), followed by maize (Figure 7a), and was less drastic in barley (Figure 7b) and tomato (Figure 7c). In addition to the quantitative increase, the high‐light grown leaves (800 and 1200 µmol m^−2^ s^−1^) of some species exhibited a shift in the reflected wavelengths from green (around 550 nm) towards yellow and red light (600–650 nm). Reflectance in the UV region (UV‐B 280–320 nm, UV‐A 320–400 nm) was generally low (< 10% in the monocots, < 5% in the dicots) and was not affected by the light intensity during plant growth.

## 4 Discussion

### 4.1 Plant Growth and Leaf Morphology in Relation to Light Quantity

An increase in PPFD (i.e., the quantity of PAR) promoted the accumulation of above‐ground biomass in both mono‐ and dicotyledonous species (Figure 2a,b). It is well established that PPFD (below stress‐inducing levels) improves plant biomass production, as it increases the net photosynthetic rate, favours the resource allocation into growth, and accordingly improves the overall photosynthetic capacity (Bruggink 1992; Dou et al. 2018; Feng et al. 2019; Ghorbanzadeh et al. 2021; Tang et al. 2022; Gonçalves et al. 2005). In the controlled environment of a growth chamber, the accumulation of dry matter was almost linearly correlated with PPFD in all species until a point of maximal convertible light energy was reached (at 800 µmol m^−2^ s^−1^ in maize, barley and bean, and at 400 µmol m^−2^ s^−1^ in tomato). Alongside the observed acceleration in growth, the shoot RWCs declined in all species (Figure 2c,d). In a well‐suited light environment, plants do not only show improved biomass production in a quantitative sense but also qualitatively as the mechanical stability of the shoot is enhanced by the deposition of solid cell components (such as lignin, cellulose or pectin) into the cell walls (Geitmann 2024). The cease of growth observed in all species under 1200 µmol m^−2^ s^−1^ compared to 800 µmol m^−2^ s^−1^ (Figure 2a,b) indicates that such intense irradiation levels induced considerable light stress (Shafiq et al. 2021).

Comparable light intensities are frequently reached in the field, however, without impairing plant development. Here, it needs to be considered that the level of sunlight outdoors varies significantly throughout the day (low in the morning and evening, peak around noon, and potential shading effects of clouds), while in the growth chamber, plants are exposed to constant levels of high‐light over the entire daily illumination phase. To facilitate the comparison of the artificial light settings to natural field conditions, the light treatments should be considered as DLI rather than irradiation level: When integrated over an illumination period of 16 h, a PPFD of 1200 µmol m^−2^ s^−1^ corresponds to a DLI of almost 70 mol m^−2^ d^−1^. In comparison, Poorter et al. (2016) reported maximum DLIs between 40 and 45 mol m^−2^ d^−1^ in the field, which were monthly averaged DLIs measured in summer in the temperate and subtropical region of the northern hemisphere. The major cultivation sites of maize, barley, tomato and bean are roughly located within this area (Food and Agriculture Organization of the United Nations, Corporate Statistical Database, https://www.fao.org/statistics/en), and it can be assumed that these DLIs approximate their respective cultivation habitats. The artificial high‐light treatment (1200 µmol m^−2^ s^−1^ or 70 mol m^−2^ d^−1^) of this study exceeds the maximum natural DLIs measured in the field (40–45 mol m^−2^ d^−1^) by 1.5‐fold, and it is not surprising that plants cultivated under such extreme light conditions, that will hardly be encountered in their natural environment, displayed visible symptoms of high‐light stress.

Evolutionary, plants developed various mechanisms to prevent oxidative stress and successive photoinhibitory effects, which include a considerable plasticity in leaf structure and physiology. While only minor changes in leaf area and LMA upon high‐light exposure were found in the monocots (Figure 2e,g), the leaf anatomy of both dicots was highly responsive to PPFD (Figure 2f,h). It appears that PPFD < 100 µmol m^−2^ s^−1^ may have induced shade avoidance, a physiological process that promotes the internode elongation at the expense of leaf expansion and thus results in small and thin leaves (Franklin 2008). When more light is available, more biomass can be allocated to leaf blade growth and mesophyll thickness to optimize light capture (Wu, Gong, and Yang 2017; Tholen, Boom, and Zhu 2012). At the same time, chlorophyll is accumulated to improve the photosynthetic productivity (Wu et al. 2021) (Figure 3b). PPFD > 400 µmol m^−2^ s^−1^ induced a subsequent reduction in the leaf surface area while they continuously became more robust (i.e., heavier) (Figure 2h) and accumulated photoprotective flavonols (Figure 3d). The small leaf area, high LMA (indicating an increased number of photosynthetic tissue layers and strengthened cell walls) and increased amounts of light‐screening pigments found under 800 and 1200 µmol m^−2^ s^−1^ are common high‐light‐induced adaptations of dicot leaves that serve to prevent photooxidative damage by excessive light energy and limit water loss through transpiration (Lefsrud et al. 2006; Dou et al. 2018; Yang et al. 2014). Monocots generally benefit from narrow and erect leaves that already reduce the amount of direct sunlight reaching the leaf surface and simultaneously minimize the transpiration area. Given that the leaf structure of grasses already displays an effective adaptation to high levels of solar irradiance, it is plausible that the anatomy was not significantly altered under conditions of high light in the growth chamber (Figure 2e,g). The only noticable response to high PPFD was the acceleration of flavonols in both monocots (Figure 3c) and an additionally decreased chlorophyll content in maize, which significantly improve the resilience in environments with intense irradiation (Ferreyra, Serra, and Casati 2021; Bian, Yang, and Liu 2015).

Shoot and leaf morphological observations combined suggest that the optimum PPFD for plant growth in dicotyledonous tomato and bean should be (i) higher than 100 µmol m^−2^ s^−1^ to supply the plant with an adequate amount of light energy but (ii) lower than 800 µmol m^−2^ s^−1^ since plants grown under 800 and 1200 µmol m^−2^ s^−1^ showed clear symptoms of oxidative stress. Nonetheless, the adaptations in leaf morphology under 800 and 1200 µmol m^−2^ s^−1^ (small, robust leaves and a high concentration of photoprotective pigments) allowed for consistent biomass production (Figure 2b) even under (solar) radiation levels that exceed the respective optimum for plant growth. Comparable light intensities have previously been suggested as the growth optimum for other dicotyledonous crops, for instance, soybean (400–500 µmol m^−2^ s^−1^; Feng et al. 2019) and lettuce (600 µmol m^−2^ s^−1^; Ghorbanzadeh et al. 2021). In contrast, the monocotyledonous maize and barley appeared to cope well even with higher irradiation levels. Based on the vegetative growth (Figure 2a), both species seem to favour PPFD of > 800 µmol m^−2^ s^−1^ as optimum growth conditions. Comparing the shoot and leaf morphology of the selected monocots to that of the dicots, it appears that maize and barley are better adapted to high levels of radiation than tomato and bean.

### 4.2 Cuticular Wax Deposition Is Promoted by Light Quantity

Our results demonstrated that the quantity of light, among all factors that constitute a plant's abiotic environment, significantly promotes the quantitative accumulation of cuticular lipids (Figure 4) without inducing major qualitative changes (Figures S1 and S2, Tables S1 and S2). This was confirmed for both monocotyledonous (maize and barley) and dicotyledonous (tomato and bean) crop species and provides supporting evidence for previous suggestions on light quantity as a driver of wax deposition (Baker 1974; Reed and Tukey 1982; Whitecross and Armstrong 1972; Nødskov Giese 1975; Kerstiens 1994; Bueno et al. 2019; Lykholat et al. 2020). However, the exact genetic mechanisms underlying light‐induced wax production are still poorly understood. Huang et al. (2020) identified some light‐responsive genes that are involved in cuticular wax biosynthesis, which might constitute promising candidates for future genetic studies on light‐regulated gene expression and wax biosynthesis. The decline in cuticular wax deposits under 1200 µmol m^−2^ s^−1^ compared to 800 µmol m^−2^ s^−1^ that was found in the dicots conceivably points towards a stress‐induced limitation of wax biosynthesis, which is supported by the morphological observations (see Section 4.1). However, further studies, including light intensities above 1200 µmol m^−2^ s^−1^ would be needed to irrevocably confirm this assumption.

Interestingly, the acceleration of wax was equally pronounced on the upper and lower leaf surfaces in almost all treatments. This is not surprising in the case of maize and barley since the anatomical and functional discrepancy between both sides of grass leaves is considerably small. However, in tomato and bean, a differential response of the light‐exposed adaxial surface and the light‐averted abaxial side was initially expected. Considering that this was not consistently observed here, it appears that, at least in certain species, PPFD drives the wax biosynthesis on a whole‐leaf level rather than exclusively in the sun‐exposed tissue layers. Moreover, it should be taken into account that the plants were only two (maize and barley) or three (tomato and bean) weeks old. Potentially, the assumed discrepancy between the upper and lower leaf surfaces might also only be established with increasing leaf age. Investigations into whether leaf age affects the adaxial/abaxial leaf wax gradient may thus be a valuable topic for future research.

### 4.3 Increased Cuticular Wax Amount Does Not Decrease the Residual (Cuticular) Transpiration

But what functional consequences come with an increased foliar wax amount, and (how) is that beneficial for the plant in a high light regime? The main function that is attributed to cuticular wax is the reduction of non‐stomatal water loss (Schönherr and Lendzian 1981). In the field, high irradiation is often associated with high temperatures and low humidity that create a vastly dehydrating environment. One might intuitively assume that higher quantities of wax produced under high PPFD serve to minimize the transpiration rate and protect the plant against desiccation. However, it has been shown repeatedly that the cuticular transpiration is independent of the amount of cuticular wax, and that already small quantities (1–1.5 µg cm^−2^) are sufficient to establish a fully functional water barrier (Schreiber and Riederer 1996; Grünhofer and Schreiber 2023; Riederer and Schreiber 2001).

**Figure 8:**
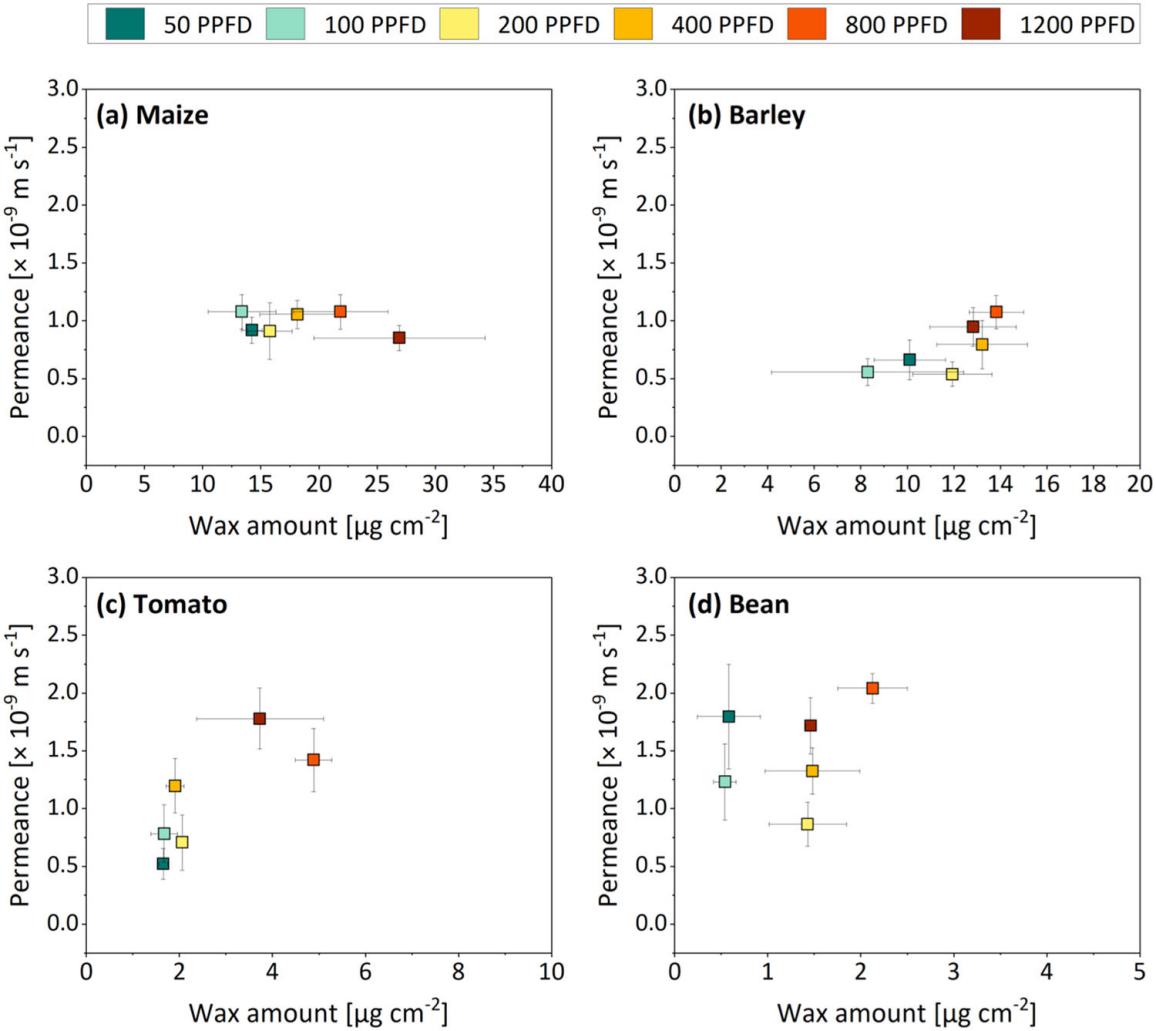
Residual permeances as a function of the cuticular wax amount of maize (a), barley (b), tomato (c) and bean (d) plants grown under six different PPFD treatments (colours are based on Figure 1). Means and standard deviations of the residual permeance are based on incremental plateauing permeances measured at a relative water deficit between 0.05 and 0.5, which was determined for each measured leaf individually (*n* = 24–40) (an example is visualized in Figure S5). Means and standard deviations of the cuticular wax amount were calculated based on the sum of adaxial and abaxial wax amounts divided by two. This was done because the transpiration occurs across both leaf sides simultaneously. PPFD, photosynthetic photon flux density. [Color figure can be viewed at wileyonlinelibrary.com]

When the residual (cuticular) permeances were plotted as a function of the total cuticular wax amount (sum of adaxial and abaxial wax amount divided by two, because transpiration occurs over both sides of the leaf simultaneously) (Figure 8), our results confirm these previous reports and could not reveal a negative correlation between the wax amount and cuticular transpiration rate (i.e., more wax does not equal lower rates of water loss). Although substantial precautions were taken in processing and evaluating the raw data (Figure S5), it must be reconsidered once more that full stomatal closure cannot be ensured with the method used. The congruence of residual and cuticular transpiration appears to be very much species‐specific with a large degree of variation (Šantrůček et al. 2004, Šantrůček 2022, Garen and Michaletz 2024), leaving us here with a highly indicative but not unequivocally confirmable set of data. While the production of cuticular wax clearly is subject to the environment (Domínguez, Cuartero, and Heredia 2011; Lewandowska, Keyl, and Feussner 2020), the cuticular water barrier appears to be largely independent of external cues (Schuster, Burghardt, and Riederer 2017; Bueno et al. 2019; Grünhofer, Herzig, Sent, et al. 2022). In a challenging environment, the plant's water status rather appears to be controlled by short‐term stomatal closure and long‐term broad‐ranging physiological and anatomical adaptions (i.e., optimization of surface‐area‐to‐volume ratio) than by modifications of the cuticular barrier properties.

### 4.4 Increased Cuticular Wax Amount Increases the Foliar Reflectance

Instead, the accumulation of wax under high light intensities may serve to increase foliar reflectance. Cuticular waxes are considered effective reflectors of both UV‐B radiation and PAR: Holmes and Keiller (2002) demonstrated a significant decrease in foliar reflectance at 330 nm (UV) and 680 nm (PAR) upon wax removal in waxy *Eucalyptus* species, while the solvent treatment of non‐waxy leaves did not change the reflectance considerably. Further, Johnson, Richards and Turner (1983) reported that the PAR reflectance of *Triticum* leaves was proportional to the amount of wax present. Here, we discovered an overall increased PAR reflectance of leaves developed under high PPFD (1200 µmol m^−2^ s^−1^) compared to leaves of the same species cultivated under low PPFD (100 µmol m^−2^ s^−1^) (Figure 7). Thus, it appears that higher wax amounts (on leaves grown under high PPFD compared to low PPFD) indeed increase the PAR reflectance, and might serve to attenuate harmful irradiation levels. However, the correlation of reflection and cuticular wax coverage is not as straightforward as one might expect. The monocots exhibited several times more wax than the dicots and additionally displayed a pronounced epicuticular wax bloom (Figure 6) that was previously suggested to increase the reflectivity (Grant et al. 2003; Grant et al. 1995). Thus, the reflection of both grass species was anticipated to be higher than that of tomato and bean leaves. No such observation was made here. Quite on the contrary, the highest reflection was measured in bean (Figure 7), the species that not only exhibited the lowest wax deposits (< 2 µg cm^−2^) but that was also devoid of prominent crystalloid epicuticular waxes (Figure 6). It is important to consider that not exclusively the cuticular reflection, but the reflection of leaves as a whole was measured. Grant (1987) stated that leaves reflect incident light in two ways; (i) by specular reflectance that takes place at the outermost surface (i.e., the cuticle) and (ii) in a diffuse manner by the leaf interior. Our results suggest that an increase in PPFD triggers morphological changes in leaves that significantly contribute to the overall reflectivity in addition to the photoprotective external wax layer.

Despite the acceleration of cuticular wax, no changes in the UV reflection were observed. Here, it needs to be considered that high‐light exposure additionally induced a significant accumulation of UV‐absorbing flavonols (Figure 3c,d). Where absorption is high, reflectance is generally low. Thus, the increased absorption of UV radiation by light‐screening pigments might compensate for the increased potential to reflect UV radiation through the accumulation of cuticular lipids. The deposition of flavonols (and the simultaneous decrease in chlorophyll) is also assumed to have induced the observed qualitative shift in reflected light from green towards yellow/red wavelengths in some species (compare Figure 7 with Figure 3).

## 5 Conclusion

The quantity of light, among all external factors that influence plant development, significantly promoted the quantitative increase in cuticular wax, while it had no effect on the qualitative lipid composition. This was observed in both monocotyledonous (maize and barley) and dicotyledonous (tomato and bean) crop species. The increase in absolute cuticular wax deposits did not decrease the residual (cuticular) transpiration, as it is often assumed mistakenly, and the results confirmed that rates of foliar water loss are indeed independent of the cuticular wax amount. Instead, the accumulation of cuticular wax in high‐light environments might rather serve to attenuate harmful irradiation levels. It was shown, however, that this relation is not straightforward and will need further investigations in future studies.

## Conflicts of Interest

The authors declare no conflicts of interest.

## Supporting information

Supporting information.

## Data Availability

The data generated during this study are available from the corresponding author upon reasonable request.
