# Supplementary material for: In a Different Light: Irradiation‐Induced Cuticular Wax Accumulation Fails to Reduce Cuticular Transpiration
Source: Plant Cell Environ. 2025 Jan 13;48(5):3632–46. doi: 10.1111/pce.15376 (PMC11963476; doi:10.1111/pce.15376)
Supplement: Supplementary file 1 — Supporting information. [file PCE-48-3632-s001.docx]

# Supplementary Figures


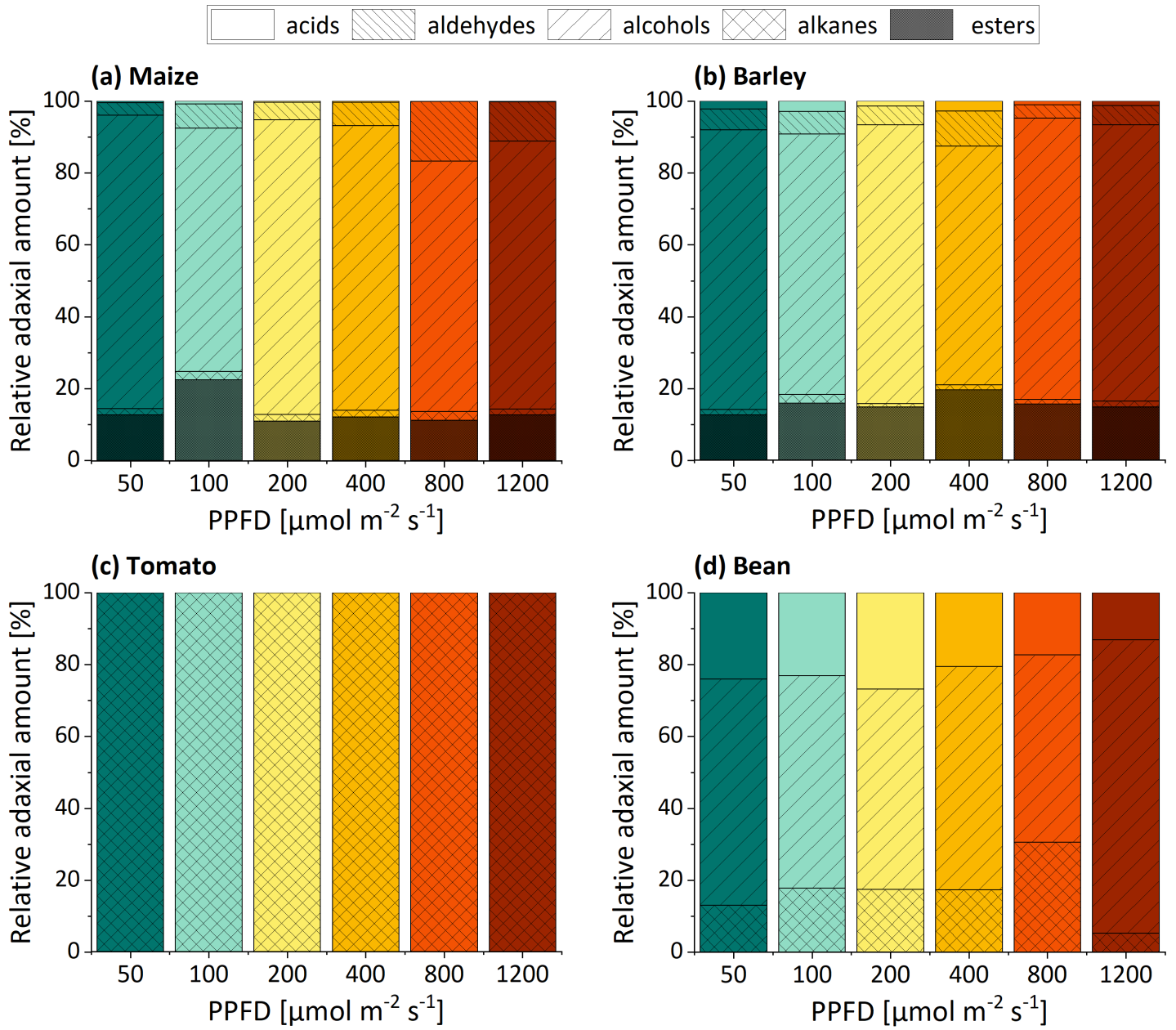


## **Figure S1:** Relative adaxial cuticular wax amount of maize (a), barley (b), tomato (c), and bean (d) grown under six different PPFD treatments (colors are based on Fig. 1). Depending on the species, the cuticular wax was composed of combinations of the functional groups acids, aldehydes, alcohols, alkanes, and esters. Means without standard deviations are shown (*n* = 3–4). The corresponding standard deviations and significance indicators are shown in Table S1. PPFD, photosynthetic photon flux density

## **Table S1:** Relative adaxial cuticular wax amount of maize, barley, tomato, and bean grown under six different PPFD treatments. Means with standard deviations are provided (*n* = 4). Arrows indicate the author’s interpretation of consistent light-dependent trends in functional group abundance across all treatments. Differential letters indicate significant differences (one-way ANOVA) within a functional group across all treatments at p ≤ 0.05. SD, standard deviation; PPFD, photosynthetic photon flux density; →, no change; ↗, increase; ↘ decrease

| PPFD (µmol m^‑2^ s^‑1^) | | 50 | | 100 | | 200 | | 400 | | 800 | | 1200 | | Trend |
| --- | --- | --- | --- | --- | --- | --- | --- | --- | --- | --- | --- | --- | --- | --- |
| Relative amount (%) | | **Mean** | **±SD** | **Mean** | **±SD** | **Mean** | **±SD** | **Mean** | **±SD** | **Mean** | **±SD** | **Mean** | **±SD** |  |
| Maize | Acids | 0.45 | 0.31 | 0.84 | 0.94 | 0.35 | 0.25 | 0.40 | 0.03 | 0.19 | 0.14 | 0.27 | 0.19 | → |
|  |  | **ab** |  | **a** |  | **ab** |  | **ab** |  | **b** |  | **ab** |  |  |
|  | Aldehydes | 3.49 | 2.20 | 6.68 | 7.05 | 4.85 | 3.25 | 6.49 | 1.27 | 16.60 | 5.64 | 10.94 | 4.31 | → |
|  |  | **a** |  | **ab** |  | **ab** |  | **ab** |  | **c** |  | **bc** |  |  |
|  | Alcohols | 81.64 | 4.22 | 67.75 | 31.40 | 82.00 | 2.47 | 79.13 | 1.48 | 69.63 | 4.39 | 74.52 | 4.17 | → |
|  |  | **a** |  | **a** |  | **a** |  | **a** |  | **a** |  | **a** |  |  |
|  | Alkanes | 1.69 | 0.35 | 2.35 | 1.87 | 1.84 | 0.07 | 1.85 | 0.65 | 2.41 | 0.21 | 1.60 | 0.21 | → |
|  |  | **a** |  | **a** |  | **a** |  | **a** |  | **a** |  | **a** |  |  |
|  | Esters | 12.72 | 2.07 | 22.38 | 21.66 | 10.95 | 1.04 | 12.12 | 2.12 | 11.17 | 2.04 | 12.68 | 2.43 | → |
|  |  | **a** |  | **a** |  | **a** |  | **a** |  | **a** |  | **a** |  |  |
| Barley | Acids | 2.26 | 0.20 | 3.80 | 2.33 | 1.36 | 0.31 | 3.18 | 1.42 | 1.03 | 0.37 | 1.27 | 1.33 | → |
|  |  | **abc** |  | **a** |  | **bc** |  | **ab** |  | **c** |  | **c** |  |  |
|  | Aldehydes | 5.61 | 1.17 | 5.67 | 3.21 | 5.16 | 1.16 | 12.83 | 11.07 | 3.75 | 0.83 | 5.10 | 3.93 | → |
|  |  | **ab** |  | **ab** |  | **a** |  | **b** |  | **a** |  | **a** |  |  |
|  | Alcohols | 77.84 | 1.21 | 69.10 | 8.51 | 77.62 | 1.21 | 56.78 | 34.12 | 78.28 | 1.50 | 77.71 | 6.60 | → |
|  |  | **a** |  | **a** |  | **a** |  | **a** |  | **a** |  | **a** |  |  |
|  | Alkanes | 1.47 | 0.51 | 3.45 | 2.36 | 1.00 | 0.05 | 1.86 | 1.52 | 1.26 | 0.16 | 1.66 | 0.56 | → |
|  |  | **a** |  | **b** |  | **a** |  | **ab** |  | **a** |  | **a** |  |  |
|  | Esters | 12.81 | 1.02 | 17.99 | 4.89 | 14.85 | 0.55 | 25.35 | 20.18 | 15.68 | 1.11 | 14.26 | 2.99 | → |
|  |  | **a** |  | **a** |  | **a** |  | **a** |  | **a** |  | **a** |  |  |
| Tomato | Acids | 0 | 0 | 0 | 0 | 0 | 0 | 0 | 0 | 0 | 0 | 0 | 0 | → |
|  |  | **a** |  | **a** |  | **a** |  | **a** |  | **a** |  | **a** |  |  |
|  | Aldehydes | 0 | 0 | 0 | 0 | 0 | 0 | 0 | 0 | 0 | 0 | 0 | 0 | → |
|  |  | **a** |  | **a** |  | **a** |  | **a** |  | **a** |  | **a** |  |  |
|  | Alcohols | 0 | 0 | 0 | 0 | 0 | 0 | 0 | 0 | 0 | 0 | 0 | 0 | → |
|  |  | **a** |  | **a** |  | **a** |  | **a** |  | **a** |  | **a** |  |  |
|  | Alkanes | 100 | 0 | 100 | 0 | 100 | 0 | 100 | 0 | 100 | 0 | 100 | 0 | → |
|  |  | **a** |  | **a** |  | **a** |  | **a** |  | **a** |  | **a** |  |  |
|  | Esters | 0 | 0 | 0 | 0 | 0 | 0 | 0 | 0 | 0 | 0 | 0 | 0 | → |
|  |  | **a** |  | **a** |  | **a** |  | **a** |  | **a** |  | **a** |  |  |
| Bean | Acids | 22.59 | 4.36 | 22.59 | 3.92 | 28.07 | 8.04 | 20.68 | 6.22 | 18.14 | 4.46 | 14.03 | 9.43 | → |
|  |  | **ab** |  | **ab** |  | **a** |  | **ab** |  | **b** |  | **b** |  |  |
|  | Aldehydes | 0 | 0 | 0 | 0 | 0 | 0 | 0 | 0 | 0 | 0 | 0 | 0 | → |
|  |  | **a** |  | **a** |  | **a** |  | **a** |  | **a** |  | **a** |  |  |
|  | Alcohols | 61.60 | 3.15 | 58.08 | 9.03 | 55.49 | 5.31 | 62.26 | 3.24 | 51.28 | 3.37 | 80.45 | 11.53 | → |
|  |  | **a** |  | **ab** |  | **ab** |  | **a** |  | **b** |  | **c** |  |  |
|  | Alkanes | 15.81 | 6.76 | 19.34 | 9.36 | 16.43 | 6.57 | 17.06 | 4.99 | 30.58 | 4.18 | 5.52 | 2.24 | → |
|  |  | **a** |  | **a** |  | **a** |  | **a** |  | **b** |  | **c** |  |  |
|  | Esters | 0 | 0 | 0 | 0 | 0 | 0 | 0 | 0 | 0 | 0 | 0 | 0 | → |
|  |  | **a** |  | **a** |  | **a** |  | **a** |  | **a** |  | **a** |  |  |


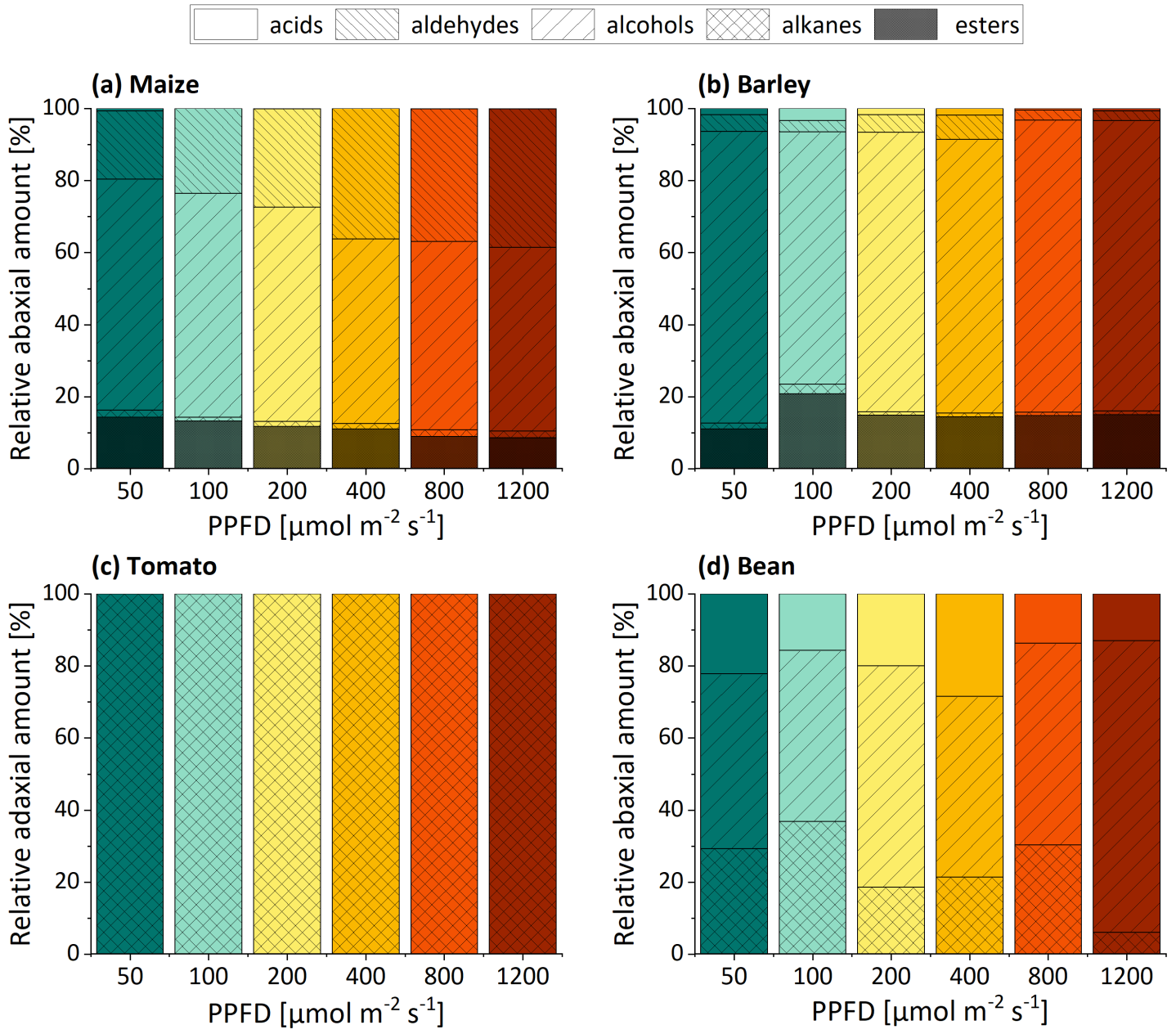


## Figure S2: Relative abaxial cuticular wax amount of maize (a), barley (b), tomato (c), and bean (d) grown under six different PPFD treatments (colors are based on Fig. 1). Depending on the species, the cuticular wax was composed of combinations of the functional groups acids, aldehydes, alcohols, alkanes, and esters. Means without standard deviations are shown (*n* = 3–4). The corresponding standard deviations and significance indicators are shown in Table S2. PPFD, photosynthetic photon flux density

## **Table S2:** Relative abaxial cuticular wax amount of maize, barley, tomato, and bean grown under six different PPFD treatments. Means with standard deviations are provided (*n* = 4). Arrows indicate the author’s interpretation of consistent light-dependent trends in functional group abundance across all treatments. Differential letters indicate significant differences (one-way ANOVA) within a functional group across all treatments at p ≤ 0.05. SD, standard deviation; PPFD, photosynthetic photon flux density; →, no change; ↗, increase; ↘ decrease

| PPFD (µmol m^‑2^ s^‑1^) | | 50 | | 100 | | 200 | | 400 | | 800 | | 1200 | | Trend |
| --- | --- | --- | --- | --- | --- | --- | --- | --- | --- | --- | --- | --- | --- | --- |
| Relative amount (%) | | **Mean** | **±SD** | **Mean** | **±SD** | **Mean** | **±SD** | **Mean** | **±SD** | **Mean** | **±SD** | **Mean** | **±SD** |  |
| Maize | Acids | 0.68 | 0.08 | 0 | 0 | 0.11 | 0.22 | 0.08 | 0.16 | 0.15 | 0.30 | 0.10 | 0.14 | → |
|  |  | **a** |  | **b** |  | **b** |  | **b** |  | **b** |  | **b** |  |  |
|  | Aldehydes | 18.97 | 3.92 | 23.65 | 5.07 | 27.26 | 6.35 | 36.14 | 1.76 | 36.84 | 8.64 | 38.49 | 4.83 | ↗ |
|  |  | **a** |  | **ab** |  | **b** |  | **c** |  | **c** |  | **c** |  |  |
|  | Alcohols | 64.10 | 4.43 | 62.05 | 4.30 | 59.46 | 7.29 | 51.23 | 2.04 | 52.16 | 7.69 | 50.90 | 5.46 | ↘ |
|  |  | **a** |  | **a** |  | **ab** |  | **bc** |  | **bc** |  | **c** |  |  |
|  | Alkanes | 1.95 | 2.04 | 1.02 | 0.27 | 1.38 | 0.19 | 1.46 | 0.24 | 1.91 | 0.75 | 1.87 | 0.58 | → |
|  |  | **a** |  | **a** |  | **a** |  | **a** |  | **a** |  | **a** |  |  |
|  | Esters | 14.30 | 1.14 | 13.28 | 1.20 | 11.79 | 2.83 | 11.08 | 1.94 | 8.94 | 2.77 | 8.63 | 2.51 | ↘ |
|  |  | **a** |  | **a** |  | **ab** |  | **ab** |  | **b** |  | **b** |  |  |
| Barley | Acids | 1.73 | 1.16 | 3.41 | 1.83 | 1.72 | 0.25 | 1.92 | 0.04 | 0.53 | 0.72 | 0.67 | 1.02 | → |
|  |  | **a** |  | **b** |  | **a** |  | **ab** |  | **a** |  | **a** |  |  |
|  | Aldehydes | 4.64 | 2.36 | 3.14 | 0.93 | 4.92 | 0.79 | 6.72 | 0.71 | 2.72 | 1.93 | 2.74 | 1.70 | → |
|  |  | **ab** |  | **a** |  | **ab** |  | **b** |  | **a** |  | **a** |  |  |
|  | Alcohols | 80.96 | 1.71 | 69.94 | 12.45 | 77.60 | 3.41 | 75.87 | 3.11 | 81.01 | 2.82 | 80.57 | 2.87 | → |
|  |  | **a** |  | **b** |  | **ab** |  | **ab** |  | **a** |  | **a** |  |  |
|  | Alkanes | 1.61 | 0.48 | 2.69 | 1.74 | 0.83 | 0.57 | 1.09 | 0.15 | 1.01 | 0.24 | 0.98 | 0.07 | → |
|  |  | **ab** |  | **a** |  | **b** |  | **b** |  | **b** |  | **b** |  |  |
|  | Esters | 11.05 | 1.08 | 20.81 | 9.12 | 14.92 | 3.15 | 14.39 | 2.56 | 14.74 | 0.68 | 15.04 | 1.13 | → |
|  |  | **a** |  | **b** |  | **ab** |  | **a** |  | **ab** |  | **ab** |  |  |
| Tomato | Acids | 0 | 0 | 0 | 0 | 0 | 0 | 0 | 0 | 0 | 0 | 0 | 0 | → |
|  |  | **a** |  | **a** |  | **a** |  | **a** |  | **a** |  | **a** |  |  |
|  | Aldehydes | 0 | 0 | 0 | 0 | 0 | 0 | 0 | 0 | 0 | 0 | 0 | 0 | → |
|  |  | **a** |  | **a** |  | **a** |  | **a** |  | **a** |  | **a** |  |  |
|  | Alcohols | 0 | 0 | 0 | 0 | 0 | 0 | 0 | 0 | 0 | 0 | 0 | 0 | → |
|  |  | **a** |  | **a** |  | **a** |  | **a** |  | **a** |  | **a** |  |  |
|  | Alkanes | 100 | 0 | 100 | 0 | 100 | 0 | 100 | 0 | 100 | 0 | 100 | 0 | → |
|  |  | **a** |  | **a** |  | **a** |  | **a** |  | **a** |  | **a** |  |  |
|  | Esters | 0 | 0 | 0 | 0 | 0 | 0 | 0 | 0 | 0 | 0 | 0 | 0 | → |
|  |  | **a** |  | **a** |  | **a** |  | **a** |  | **a** |  | **a** |  |  |
| Bean | Acids | 18.52 | 8.20 | 13.08 | 9.42 | 19.86 | 1.72 | 27.69 | 7.49 | 13.88 | 5.48 | 12.24 | 6.49 | → |
|  |  | **ab** |  | **a** |  | **ab** |  | **b** |  | **a** |  | **a** |  |  |
|  | Aldehydes | 0 | 0 | 0 | 0 | 0 | 0 | 0 | 0 | 0 | 0 | 0 | 0 | → |
|  |  | **a** |  | **a** |  | **a** |  | **a** |  | **a** |  | **a** |  |  |
|  | Alcohols | 44.49 | 9.20 | 44.06 | 9.89 | 63.86 | 10.63 | 55.05 | 14.55 | 55.80 | 6.00 | 81.87 | 7.71 | ↗ |
|  |  | **a** |  | **a** |  | **b** |  | **bc** |  | **bc** |  | **c** |  |  |
|  | Alkanes | 36.99 | 17.19 | 42.86 | 19.02 | 16.28 | 10.69 | 17.26 | 11.28 | 30.32 | 2.48 | 5.90 | 1.25 | ↘ |
|  |  | **a** |  | **a** |  | **bc** |  | **bc** |  | **ab** |  | **c** |  |  |
|  | Esters | 0 | 0 | 0 | 0 | 0 | 0 | 0 | 0 | 0 | 0 | 0 | 0 | → |
|  |  | **a** |  | **a** |  | **a** |  | **a** |  | **a** |  | **a** |  |  |


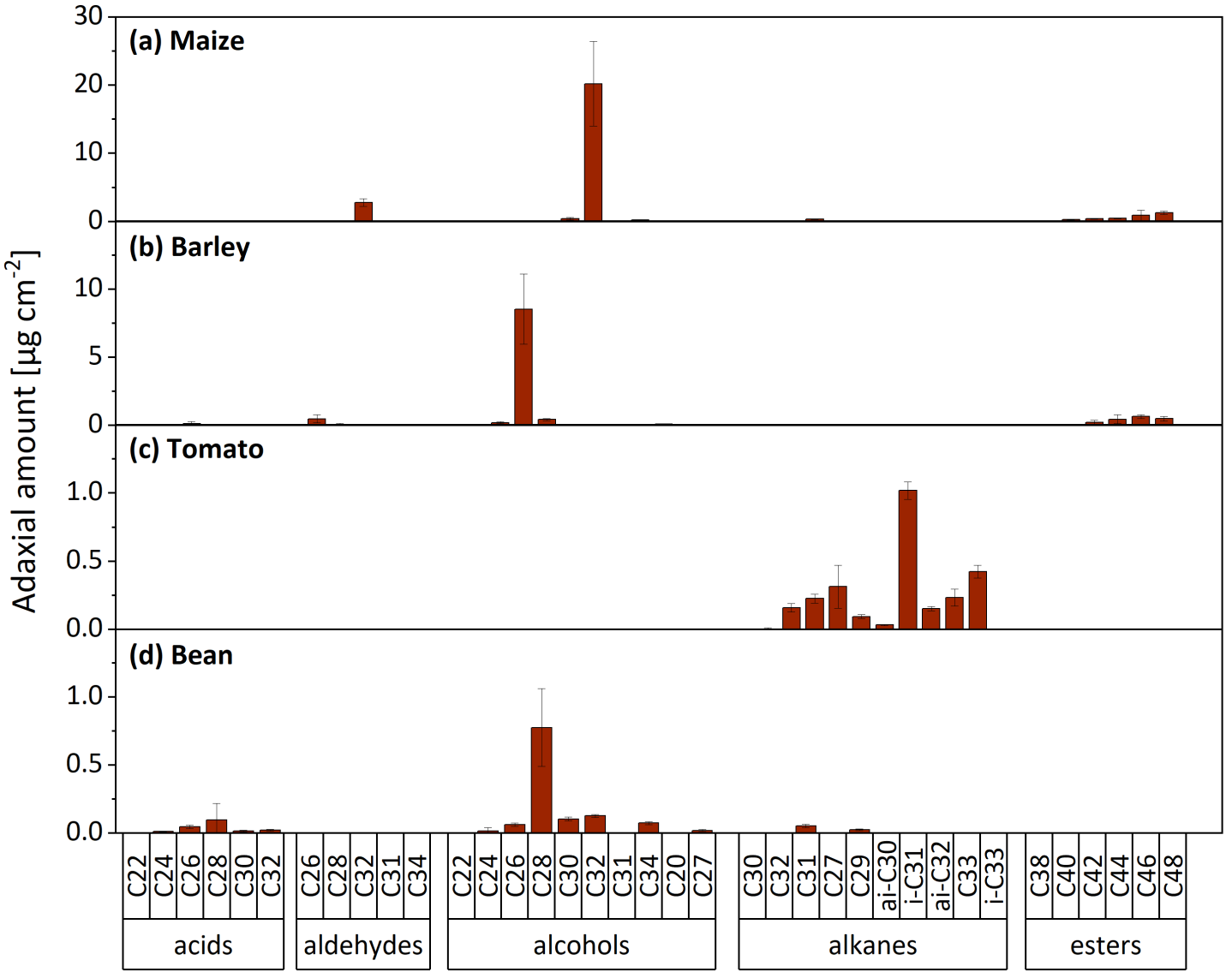


## **Figure S3:** Representative monomer composition of the adaxial cuticular wax of maize (a), barley (b), tomato (c), and bean (d) grown under 1200 µmol m^‑2^ s^‑1^. Depending on the species, the cuticular wax was composed of combinations of the functional groups acids, aldehydes, alcohols, alkanes, and esters. Means with standard deviations are shown (*n* = 3–4)


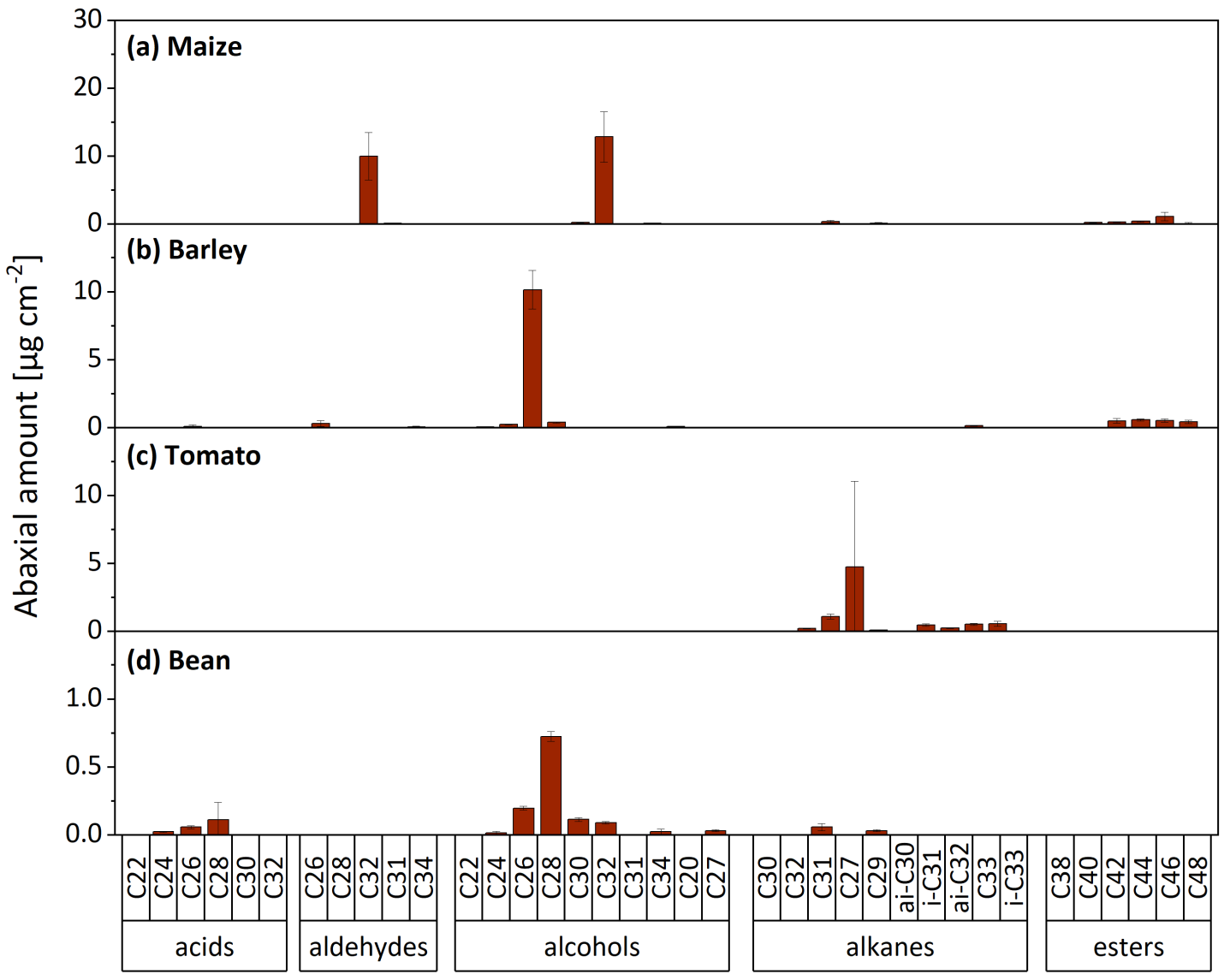


## **Figure S4:** Representative monomer composition of the abaxial cuticular wax of maize (a), barley (b), tomato (c), and bean (d) grown under 1200 µmol m^‑2^ s^‑1^. Depending on the species, the cuticular wax was composed of combinations of the functional groups acids, aldehydes, alcohols, alkanes, and esters. Means with standard deviations are shown (*n* = 3–4)


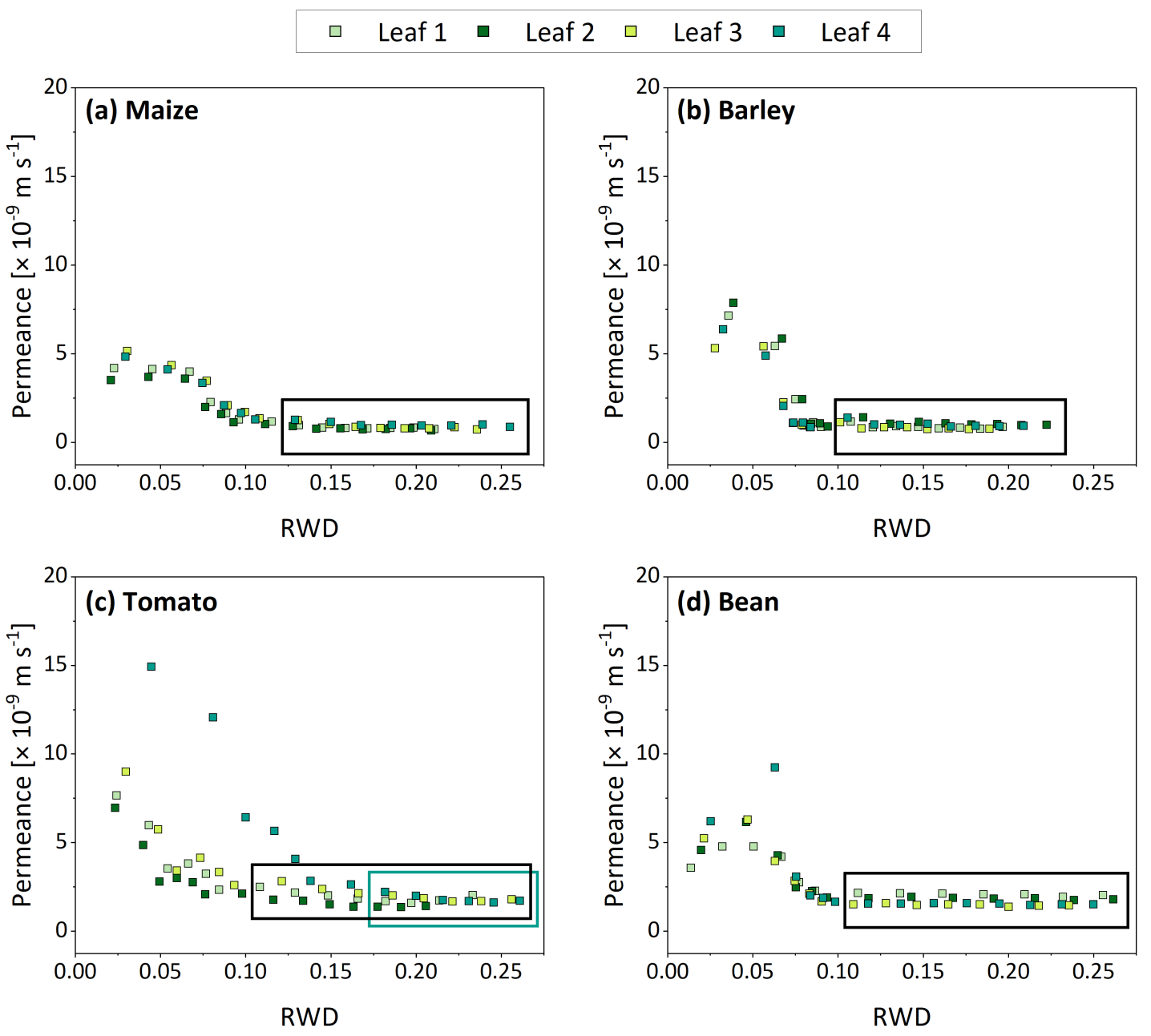


## **Figure S5:** Incremental permeances of detached leaves of maize (a), barley (b), tomato (c), and bean (d) plants plotted against the relative water deficit. Representative plots obtained from the 1200 µmol m^‑2^ s^‑1^ treatment are shown. Plateauing incremental permeances during the period of maximum stomatal closure (indicated by squares) were selected and pooled to calculate the final residual permeances. Given that stomata closed at different relative water deficits depending on the leaf (e.g., additional green square in the case of tomato), the plateauing permeances were selected for each leaf individually. RWD, relative water deficit
